# Light-triggered carbon monoxide-induced activation of enhanced ferritinophagy-mediated ferroptosis for bone metastases therapy

**DOI:** 10.1016/j.mtbio.2025.102322

**Published:** 2025-09-17

**Authors:** Qin Liu, Jian Zhang, Lujie Yu, Yaohua Chen, Chiyv Zhang, Juan Li, Shutong Wu, Xiaochun Zheng, Rong Dai, Ziliang Zheng, Ruiping Zhang

**Affiliations:** a Shanxi Bethune Hospital, Shanxi Academy of Medical Third Hospital of Shanxi Medical University, Tongji Sciences, Hospital, Taiyuan, 030032, China; b Laboratory of Molecular Imaging, Fifth Hospital of Shanxi Medical University (Shanxi Provincial People's Hospital), Taiyuan, 030000, China

**Keywords:** Second near-infrared fluorescence imaging, Carbon monoxide, Photocatalysis, Ferroptosis, Bone metastases

## Abstract

Bone metastases, as a common disabling and life-threatening complication in the advanced stages of various solid tumors, continue to pose substantial therapeutic challenges due to high drug toxicity and tumor resistance. To overcome the limited efficacy and safety concerns of existing treatments, we developed a novel iron-based photocatalytic nanoplatform (ENCF), guided by second near-infrared (NIR-II) imaging, for the precise treatment of bone metastases. This platform enables in situ photocatalytic release of CO and utilizes exposed iron active sites to synergistically induce ferroptosis through a cascade of oxidative stress, autophagy and iron metabolism disruption under 808 nm laser activation. Mechanistic investigations revealed that the ENCF platform significantly downregulates PCBP2, a key regulator of ferritinophagy, while activating LC3- and ATG5-mediated autophagic pathways to accelerate FTH1 degradation and Fe^2+^ release, thereby disturbing intracellular iron homeostasis. Concurrently, the released CO disrupts mitochondrial electron transport and inhibits ATP synthesis, leading to excessive ROS accumulation, enhanced suppression of GPX4 , accelerated lipid peroxidation, and the initiation of a robust ferroptotic response. Benefiting from its deep-tissue photoactivation, high catalytic efficiency, and multi-target synergistic mechanisms, ENCF achieved potent tumor suppression with selective accumulation at metastatic sites in a bone metastasis model. Collectively, this study establishes a multi-pronged therapeutic strategy via “CO release–autophagy enhancement–ferroptosis activation,” offering a promising and innovative approach for the precise treatment of bone metastases.

## Graphical abstract

In this work, we developed a NIR-II fluorescence–guided photocatalytic nanoplatform that triggers ferroptosis via CO/Fe synergy for precise diagnosis and treatment of bone metastases.

**Figure:**
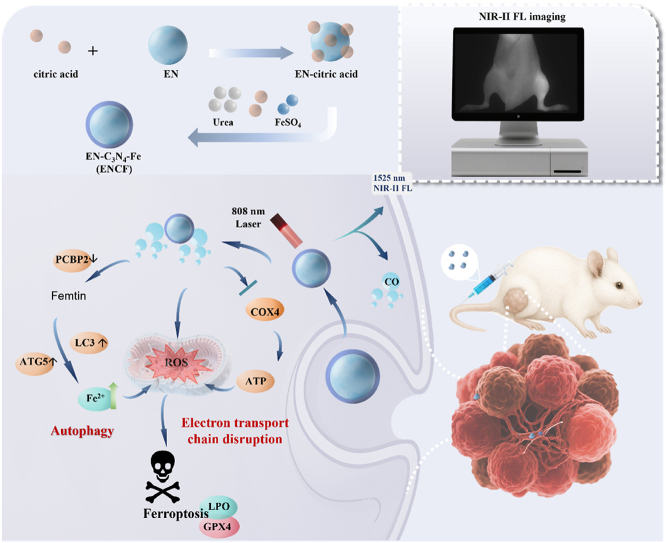

## 1 Introduction

Bone metastases (BM) [1,2] represent one of the most common forms of distant metastasis in the advanced stages of various solid tumors, including breast cancer, prostate cancer and lung cancer. Clinically, BM is often accompanied by severe complications such as intense bone pain [3], pathological fractures [4], hypercalcemia [5] and spinal cord compression [6], all of which result in high disability and mortality rates, severely impairing patients' quality of life and overall survival. Although current treatments, including chemotherapy, targeted therapies and bone-protective agents [7], offer some efficacy, their impact is limited by modest response rates and safety concerns. Chemotherapy, for instance, achieves only an 18–42 % objective response rate (ORR) and is frequently accompanied by grade ≥3 toxicities. Targeted therapies suffer from insufficient data on progression-free survival (PFS), ORR and safety, while bone-protective agents, such as denosumab, fail to induce objective tumor responses and carry risks of osteonecrosis of the jaw (5 %) and hypocalcemia (7 %) [8,9]. These limitations are especially pronounced in metastatic lesions located in deep-seated sites such as the bone marrow, where therapeutic access and control remain formidable challenges [10,11]. Given these obstacles, there is a pressing need for precision therapeutic strategies that combine deep tissue penetration, high specificity and minimal systemic toxicity. Precision therapy [12,13] has become an important strategy for addressing deep-seated tumors. In this context, exogenously activated therapeutic modalities [[14], [15], [16]], particularly those guided by second near-infrared window (NIR-II, 1000–1700 nm) fluorescence imaging [17,18] have attracted considerable attention. These techniques offer excellent spatial control, deep tissue penetration and low systemic toxicity, making them highly promising for precise intervention in deep bone metastases. This facilitates the development of an integrated “diagnosis–localization–therapy” paradigm [19], which is highly advantageous for precision management of deep bone lesions.

Among emerging therapeutic mechanisms, ferroptosis [20,21]—a novel form of regulated cell death driven by iron-dependent lipid peroxidation—has demonstrated unique potential in overcoming drug resistance. Mechanistically, intracellular ferrous ions (Fe^2+^) react with hydrogen peroxide (H_2_O_2_) through the Fenton reaction [22], generating highly reactive hydroxyl radicals (•OH). These radicals accelerate lipid peroxidation, compromise membrane integrity and ultimately induce cell death. Moreover, ferroptosis is tightly linked to the inactivation of glutathione peroxidase 4 (GPX4) [23,24], resulting in the collapse of the antioxidant defense system and exacerbating oxidative stress, redox imbalance and programmed cell death. Recent studies further highlight that functional gas molecules such as nitric oxide (NO) [25,26] and carbon monoxide (CO) [27,28] can act as powerful modulators of ferroptosis. Incorporating gas-based regulation not only broadens the therapeutic window but also enhances depth of action, selectivity and overall treatment efficacy. Such gas-augmented ferroptosis strategies provide a promising frontier for designing next-generation therapies capable of addressing the unique challenges of metastatic bone tumors.

In addition, ferritinophagy—a selective form of autophagy that degrades intracellular ferritin (FTH1)—plays a pivotal role in maintaining iron homeostasis and strongly influences both the sensitivity and intensity of ferroptosis [29,30]. By degrading FTH1, ferritinophagy liberates free Fe^2+^, fueling the Fenton reaction, amplifying lipid peroxidation, and sustaining ferroptotic signaling. However, under mild stress conditions, tumor cells often activate protective autophagy to alleviate oxidative stress, thereby weakening the ferroptotic response and contributing to therapeutic resistance [31]. Consequently, precise modulation of ferritinophagy is essential to disrupt iron equilibrium and overcome resistance, thereby maximizing ferroptosis efficacy. Previous studies have demonstrated that heat stimuli [32] or ROS-rich microenvironments [33] can induce the upregulation of key autophagic proteins such as Microtubule-associated proteins 1A/1B light chain 3 (LC3) and Autophagy-related protein 5 homolog (ATG5), initiating the ferritinophagy process [34,35]. This accelerates FTH1 degradation, increases Fe^2+^ availability and strengthens ferroptotic cascades. Building upon this, the rational design of nanoplatforms incorporating ferritinophagy activation modules [36] can establish a self-reinforcing feedback loop of “iron release–ROS amplification–GPX4 depletion,” ultimately enhancing ferroptosis and producing robust antitumor effects.

**Scheme 1:**
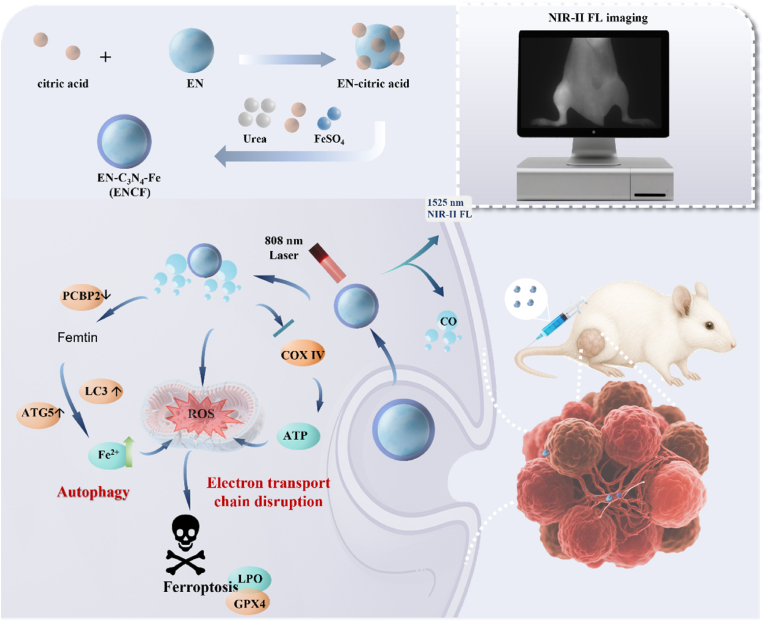
Schematic illustration of the synthesis and application of ENCF in bone metastasis treatment.

In this study, leveraging the pathological characteristics of the bone metastatic microenvironment—namely enhanced vascular permeability and heightened oxidative stress—we developed a NIR-II fluorescence imaging–guided photocatalytic nanoplatform (NaYF_4_:18 %Yb, 2 %Er@NaYF_4_:20 %Nd@C_3_N_4_-Fe, abbreviated ENCF) that integrates diagnostic and therapeutic functions (Scheme 1). The platform consists of a NaYF_4_:18 %Yb, 2 %Er@ NaYF_4_:20 %Nd (EN) coated with carbon nitride dots (C_3_N_4_), further functionalized with iron to boost catalytic performance. Under 808 nm laser irradiation, ENCF enables controllable CO release, which disrupts mitochondrial electron transport, suppresses ATP synthesis, induces mitochondrial dysfunction and increases endogenous ROS levels. Simultaneously, the iron component catalyzes a Fenton-like reaction with H_2_O_2_ to generate highly reactive •OH radicals, thereby aggravating oxidative damage. Moreover, ENCF activates the ferritinophagy pathway by downregulating the iron chaperone Poly(rC)-binding protein 2 (PCBP2) and upregulating autophagic markers LC3 and ATG5, which together accelerate FTH1 degradation, thereby liberating stored iron and increasing the labile Fe^2+^ pool. This cascade drives lipid peroxidation, disrupts cellular redox balance, impairs antioxidant defenses and ultimately induces in ferroptotic cell death. Through NIR-II fluorescence imaging, ENCF achieves precise localization of bone metastatic lesions, while laser activation triggers simultaneous CO release, ferritinophagy activation, and ferroptosis amplification, effectively suppressing tumor progression and mitigating bone destruction. This study demonstrates the platform's strong therapeutic potential and translational prospects for clinical application in bone metastases.

## 2 Results and discussion

### 2.1 Synthesis and characterization of ENCF nanoplatform

**Fig. 1:**
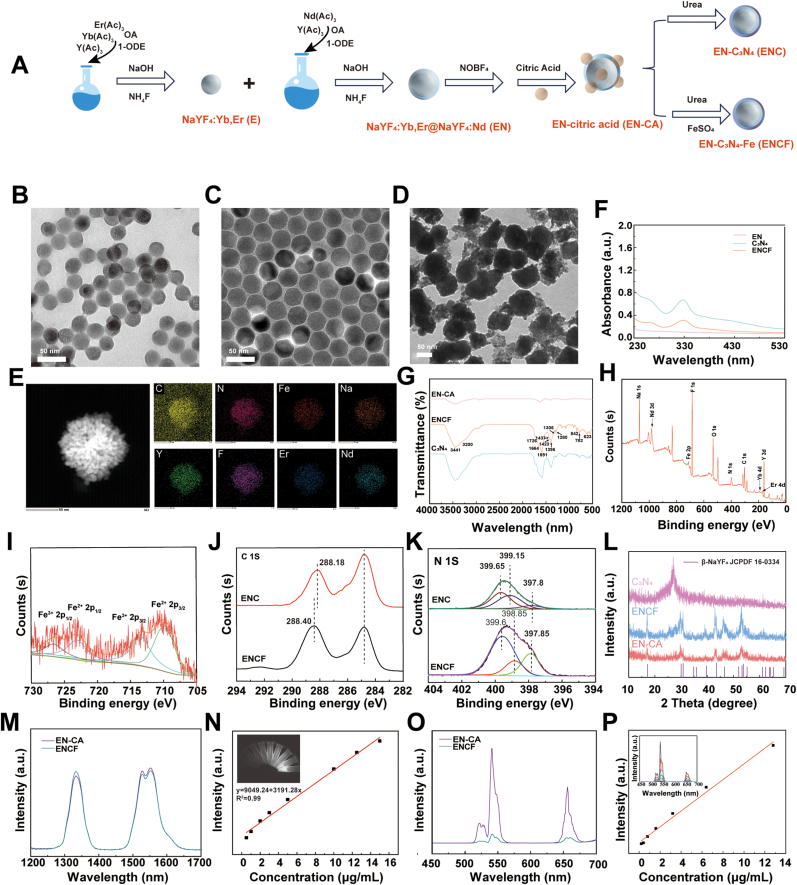
(A) Schematic illustration of ENCF synthesis. TEM of (B) E, (C) EN and (D) ENCF nanoplatform; (E) EDS elemental mappings of ENCF; (F) UV–Vis absorption spectra of EN-CA, C_3_N_4_ and ENCF; (G) FT-IR spectra of EN-CA, C_3_N_4_ and ENCF; (H) XPS survey spectra of ENCF; High-resolution XPS spectra of (I) Fe 2p, (J) C 1s and (K) N 1s regions of ENCF and ENC; (L) XRD patterns of EN-CA, C_3_N_4_ and ENCF; (M) Down-conversion fluorescence spectra of EN-CA and ENCF under 808 nm excitation; (N) NIR-II fluorescence intensity of ENCF at different concentrations and corresponding fluorescence imaging; (O) Up-conversion fluorescence spectra of EN-CA and ENCF under 808 nm excitation. (P) Standard calibration curve of ENCF upconversion fluorescence at varying concentrations (0–13 μg/mL) with corresponding emission spectra.

To realize efficient synergy between NIR-II fluorescence imaging and in situ photocatalytic therapy, we developed a multifunctional heterostructured ENCF nanoplatform. As illustrated in the synthetic schematic (Fig. 1A), the construction of ENCF followed a well-defined stepwise process. First, NaYF_4_:Yb,Er (E) nanocrystals were synthesized via a solvothermal method, which then served as the seed for the epitaxial growth of a NaYF_4_:Nd shell to yield the core–shell NaYF_4_:Yb,Er@NaYF_4_:Nd (EN). To improve surface functionality and dispersibility, EN was further modified with NOBF_4_ and citric acid to obtain citric acid-capped [37] EN (EN–CA). On this basis, two parallel hydrothermal reactions were performed: (i) EN–CA was combined with citric acid and urea to generate EN–C_3_N_4_ (ENC) [38] and (ii) EN–CA was reacted with citric acid, urea and FeSO_4_ at 180 °C for 3 h to obtain EN–C_3_N_4_–Fe (ENCF). This parallel synthetic route enables the simultaneous construction of a control material (ENC) and the functionalized nanoplatform (ENCF), as depicted in Fig. 1A. Transmission electron microscopy (TEM) clearly demonstrated the sequential morphological evolution: the pristine E nanocrystals exhibited a uniform near-spherical morphology with an average diameter of 23.4 nm (Fig. 1B and Fig. S1), and the diameter increased to 30.2 nm after the growth of the NaYF_4_:Nd shell, confirming the formation of the EN core–shell structure (Fig. 1C). Upon subsequent coating with C_3_N_4_ or C_3_N_4_–Fe, the nanoparticles further increased in size and displayed roughened surfaces, indicative of successful outer-layer assembly. Elemental mapping analysis clearly demonstrated the uniform and overlapping distribution of C, N, Fe, Na, Y, Er and Nd within the ENCF nanoplatform, confirming the successful integration of both the catalytic (C_3_N_4_–Fe) and EN luminescent components. The homogeneous spatial distribution of these elements provides direct evidence of stable structural assemblies and intimate interfacial coupling between the core and shell domains. In parallel, Zeta potential measurements tracked the surface charge evolution throughout the synthesis: the pristine EN exhibited a positive potential, which shifted to +4.51 mV after citric acid modification, and further decreased to −19.15 mV after C_3_N_4_–Fe coating, consistent with the introduction of negatively charged –COOH groups (Fig. S2) [39]. The successful construction of the shell was further supported by spectral analysis. UV–Vis absorption spectra (Fig. 1F) revealed that ENCF exhibited characteristic absorption peaks of C_3_N_4_ materials at 268 nm and 335 nm. The 268 nm peak [40] corresponds to π–π∗ transitions associated with s-triazine ring-containing carbon nitride compounds, consistent with previous reports on carbon nitride quantum dots. The 335 nm peak [[41], [42], [43], [44]] reflects the typical light absorption behavior of C_3_N_4_ semiconductors. Oxygen-containing groups introduced by citric acid may have extended the conjugated system and altered the surface electronic structure, thereby modulating both optical absorption and surface states of the C_3_N_4_ quantum dots.

Fourier-transform infrared (FT-IR) spectra (Fig. 1G) clearly display multiple absorption peaks corresponding to the characteristic vibrational modes of the C_3_N_4_ framework. The absorption bands in the range of 1200–1700 cm^−1^ are attributed to the stretching vibrations of –C=N and C–N bonds [45], which is indicative of aromatic CN heterocyclic structures. A distinct peak at 782 cm^−1^ is assigned to the breathing mode of the s-triazine ring [39], representing a key fingerprint region of C_3_N_4_. In addition, strong absorption bands observed at 1726 cm^−1^ correspond to the asymmetric stretching vibrations of C=O groups, while the broad absorptions centered at 3200 and 3441 cm^−1^ are ascribed to the stretching vibrations of N–H and O–H bonds, suggesting the potential presence of surface amino and hydroxyl functional groups. To further elucidate the elemental composition and chemical states of ENCF, X-ray photoelectron spectroscopy (XPS, Fig. 1H) confirmed the presence of Fe, C, N, Er and Nd. To further clarify the oxidation states of iron in ENCF, high-resolution XPS analysis of the Fe 2p region was performed. The deconvoluted peaks at 709.9 eV (Fe 2p_3/2_) and 723.1 eV (Fe 2p_1/2_) were assigned to Fe^2+^, while peaks at 713.3 eV and 726.8 eV were attributed to Fe^3+^, in agreement with previous reports [46]. The Fe 2p_3_/_2_ envelope was fitted with a χ^2^ value of 1.68, indicating a reliable result consistent with the XPS data [47]. The calculated Fe^2+^/Fe^3+^ area ratio was approximately 1.5, indicating that Fe^2+^ species are more abundant than Fe^3+^ in ENCF. This result was further validated by the Prussian Blue assay [48], which yielded a Fe^2+^/Fe^3+^ ratio of 1.58 (Fig. S3), highly consistent with the XPS-derived value. High-resolution C 1s and N 1s spectra (Fig. 1I–K) displayed slight binding energy shifts compared to ENC, implying that Fe was likely embedded via Fe–N or Fe–C coordination. The X-ray diffraction (XRD) pattern (Fig. 1L) clearly demonstrated that ENCF consisted of crystalline β- NaYF_4_, with the diffraction peaks well matched to the standard JCPDF card No. 16–0334, confirming the successful incorporation of the rare-earth host lattice. A characteristic diffraction peak at 27.3 further validated the presence of C_3_N_4_ [38].

With respect to optical properties, ENCF exhibited strong NIR-II downconversion luminescence under 808 nm excitation (Fig. 1M), with an emission peak at 1525 nm that closely matched with that of EN-CA. This indicates that the outer C_3_N_4_–Fe shell did not significantly interfere with the energy levels or luminescence mechanism of the EN. The absolute quantum yield of ENCF powders at 1525 nm was determined to be 3.59 %. Furthermore, the NIR-II fluorescence intensity increased in a concentration-dependent manner (Fig. 1N), and fluorescence imaging demonstrated its potential utility for NIR-II-based bioimaging. Notably, the upconversion luminescence was significantly quenched, likely due to efficient energy transfer from the excited EN-CA to the C_3_N_4_–Fe shell. Moreover, the upconversion luminescence intensity (Fig. 1P) showed a strong linear correlation with particle concentration (R^2^ = 0.99). In summary, these findings confirm the successful construction of the ENCF heterostructured core–shell nanoplatform through systematic characterization encompassing synthesis, morphology, surface charge, elemental states and optical properties.

### 2.2 Evaluation of the laser-triggered CO release and hydroxyl radical generation by ENCF

**Fig. 2:**
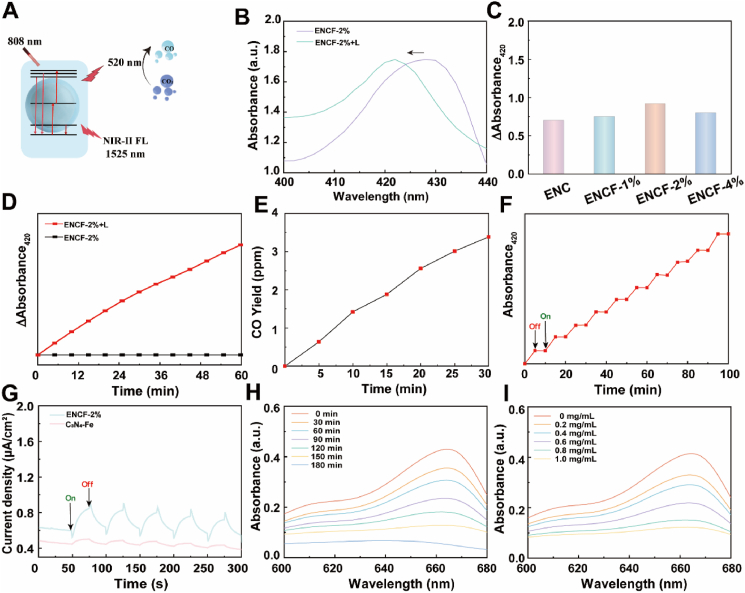
(A) Schematic illustration of ENCF under 808 nm laser irradiation leading to upconversion activation and CF-mediated CO release; (B) Absorbance change at 420 nm in HbCO after 808 nm laser irradiation (1 W/cm^2^) of ENCF-2 % solution; (C) Absorbance variation at 420 nm for ENCF with different Fe doping levels; (D) Time-dependent CO release from ENCF-2 % with or without 808 nm laser irradiation; (E) CO release behavior under alternating laser on/off cycles; (F) CO evolution over ENCF; (G) Photocurrent responses of ENCF and C_3_N_4_-Fe under repeated laser on/off cycles, reflecting photo-induced charge separation performance; (H) Time-course degradation of MB with ENCF and H_2_O_2_; (I) MB degradation with different concentrations of ENCF in H_2_O_2_ solution.

To address the complex tumor microenvironment and therapeutic challenges associated with deep-seated tumors, the ENCF platform was engineered to integrate an EN nanocore with a C_3_N_4_–Fe catalytic shell, thereby enabling multi-mechanistic synergistic therapy under near-infrared (NIR) laser activation. As illustrated in Fig. 2A, ENCF simultaneously emits both green light (520 nm) and NIR-II fluorescence (1525 nm) under 808 nm laser irradiation. The catalytic layer promoted the photocatalytic reduction of CO_2_ to CO and generated hydroxyl radicals (•OH) through Fenton-like processes. In this configuration, the EN serves as an excitation source, while the C_3_N_4_–Fe shell functions as the catalytic interface, enabling efficient excitation-to-catalysis energy transfer. To achieve quantitative detection of CO, hemoglobin (Hb) was employed as a sensing probe due to its ability to form carbonylhemoglobin (HbCO) upon binding CO (Fig. S4), which exhibits a characteristic absorbance peak at 420 nm [49]. As shown in Fig. 2B, ENCF under laser irradiation exhibited a marked increase in absorbance at 420 nm, confirming efficient CO release triggered by NIR activation and verifying the photocatalytic responsiveness of the platform. To further assess the influence of Fe doping on catalytic efficiency, ENCF nanomaterials with varying Fe contents were compared (Fig. 2C). Among them, ENCF-2 % (Fe-to-total mass ratio of 2 %) showed the highest CO release, with a 420 nm absorbance differential of 0.97, outperforming ENCF-1 % (0.75) and ENCF-4 % (0.81) by approximately 29 % and 19 %, respectively. These findings suggest that a moderate Fe doping level yields the most uniform distribution of catalytic sites. Fig. 2D shows that the CO release from ENCF-2 % increased linearly with irradiation time over 60 min (R^2^ = 0.987). This result highlights excellent catalytic durability and stability, which are critical attributes for sustained therapeutic gas generation. Furthermore, the absolute CO yield from ENCF under 808 nm laser irradiation was quantified using a conventional GC method (Fig. 2E). After subtraction of the background signal arising from CO_2_ photoreduction, the CO production rate of ENCF was determined to be 65 μmol·h^−1^·g^−1^. The temporal control of gas release was further validated via laser on–off cycling experiments (Fig. 2F), in which the absorbance at 420 nm responded rapidly to each laser switch. This finding underscores the precise spatiotemporal controllability of ENCF-mediated CO release, reinforcing its potential safety and tunability for therapeutic applications. To probe the electron transfer behavior during photoactivation, photoelectrochemical measurements were conducted (Fig. 2G) [50]. ENCF-2 % exhibited stable photocurrent responses across five on–off cycles, with an average photocurrent of 0.04 μA/cm^2^, approximately 6.5-fold higher than that of the C_3_N_4_-Fe control group (0.006 μA/cm^2^). These results indicate that the EN core enhances charge separation efficiency and promotes rapid migration of photoexcited electrons to the C_3_N_4_-Fe shell, thereby accelerating photocatalytic activity and improving overall energy utilization.

The Fenton-like reaction is a crucial component of ENCF's catalytic function, in which Fe^2+^ reacts with endogenous H_2_O_2_ in the tumor microenvironment to generate •OH. These highly reactive species not only induce ferroptosis but also activate multiple ROS-mediated signaling pathways, thereby amplifying therapeutic efficacy. To indirectly evaluate •OH generation, a methylene blue (MB) degradation assay was conducted (Fig. 2H) [51]. ENCF-2 % reduced the MB absorbance from 0.43 to 0.05 over 180 min, demonstrating sustained •OH release capacity. As shown in Fig. 2I, MB degradation efficiency increased in a concentration-dependent manner, achieving 72 % at 1.0 mg/mL, which highlights the platform's tunable ROS production and dose-responsive catalytic performance. The intimate core–shell interaction between EN and C_3_N_4_–Fe ensures efficient energy transfer from the optical absorption layer to the catalytic interface, forming the mechanistic basis for an integrated “imaging–catalysis–therapy” paradigm.

### 2.3 Inhibition of 4 T1 cells activity by ENCF

**Fig. 3:**
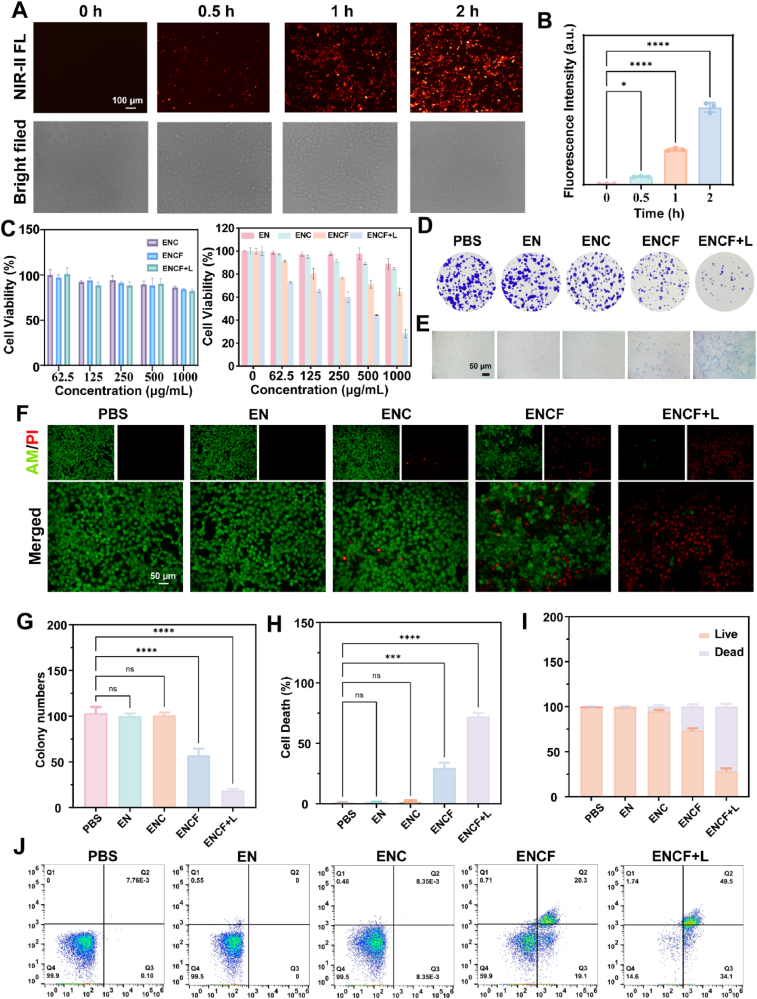
(A) Cellular uptake of ENCF in 4T1 cells after incubation for 0, 0.5, 1 and 2 h (excitation: 808 nm); (B) Quantification of cellular fluorescence intensity at different time points; (C) Cell viability of HUVEC and 4T1 cells incubated with various concentrations (0–1000 μg/mL) of different materials for 24 h, assessed by CCK-8 assay; (D) Comparison of colony formation ability in different treatment groups over 7 days; (E) Trypan blue staining of 4T1 cells in each treatment group; (F) Live/dead staining images using Calcein-AM (green, live cells) and PI (red, dead cells) following treatment with different formulations. Scale bar: 50 μm. (G–I) Quantification of Cell cloning, trypan blue and AM/PI staining in different treatment groups (n = 3); (J) Flow cytometry demonstrated that ENCF induces apoptosis in 4T1 cells.

To systematically evaluate the NIR-II imaging capability, biosafety and laser-activated synergistic therapeutic potential of the ENCF nanoplatform at the cellular level, highly invasive 4T1 breast cancer cells were selected as the in vitro model. A series of multidimensional functional assays including cellular uptake kinetics, cell viability analysis, colony formation and membrane integrity staining were conducted. Dynamic uptake of ENCF was first visualized by NIR-II fluorescence imaging. As shown in Fig. 3A and B, the intracellular fluorescence intensity at 1525 nm progressively increased over 0–2 h, confirming efficient internalization and strong imaging capability. Bright-field images revealed intact cellular morphology, with well-defined boundaries and no evidence of cytoplasmic granulation or nuclear-cytoplasmic dissociation, suggesting minimal structural perturbation under the tested conditions. To assess cytocompatibility with normal cells, CCK-8 assays were conducted using human umbilical vein endothelial cells (HUVECs). ENCF exposure at therapeutic concentrations did not significantly impair viability, supporting its favorable safety profile (Fig. 3C). To further assess cytotoxicity, a dose-dependent CCK-8 assay was conducted. Across the 0–1000 μg/mL range, EN and ENC groups maintained high viability (89.4 % and 84.7 % at 1000 μg/mL), confirming intrinsic biosafety. In contrast, ENCF induced a gradual viability decline, falling below 80 % at 250 μg/mL and reaching 62 % at 1000 μg/mL, attributable to Fe-catalyzed ROS production via Fenton-like reactions. Upon laser irradiation (ENCF + L), viability further decreased to 28 %, demonstrating a pronounced synergistic cytotoxic effect.

Long-term proliferation was examined via colony formation assays (Fig. 3D). No significant differences were observed among PBS, EN and ENC groups (Fig. 3G). ENCF treatment slightly reduced colony count, while ENCF + L induced a sharp decrease, confirming that photonic activation significantly impairs clonogenic survival. Mechanistic insights were obtained through membrane integrity analysis using trypan blue staining (Fig. 3E) [52]. The PBS, EN and ENC groups exhibited negligible positive staining (Fig. 3H). A small proportion of stained cells was observed with ENCF, while ENCF + L induced substantial membrane permeabilization, indicative of lethal membrane damage.

To further validate the mode of cell death, Calcein-AM**/**PI double staining was performed (Fig. 3F). PI fluorescence intensity in cells of the ENCF + L group was far higher than the Calcein-AM signal (Fig. 3I). Composite fluorescence imaging further demonstrated strong PI-positive clusters, indicating collective cell death, supporting the hypothesis that the ROS/CO dual mechanism drives a synergistic cytotoxic cascade. Fluorescence microscopy revealed extensive clustered cell death with blurred boundaries and irregular morphology—indicative of severe, irreversible membrane damage. In contrast, the ENCF group without irradiation showed approximately 30 % PI-positive cells, whereas the PBS, EN and ENC groups predominantly exhibited green, viable cells. Flow cytometry further substantiated these observations, revealing that over 83 % of cells in the ENCF + L group were PI-positive with a markedly diminished Calcein-AM signal, whereas the ENCF group exhibited approximately 39 % PI-positive cells and the PBS, EN and ENC groups showed less than 5 % (Fig. 3J). Above all, these results confirm that the laser-triggered release of CO and •OH from ENCF induces synergistic cytotoxicity in tumor cells with high specificity and efficacy. In summary, the results in Fig. 3 establish a comprehensive evaluation loop spanning efficient cellular uptake and NIR-II imaging, biocompatibility assessment, proliferation inhibition, membrane disruption and mechanistic elucidation of cell death. Notably, the NIR-II fluorescence-guided and controllable CO release confers a strategic advantage for penetrating the tumor microenvironment and inducing ferroptosis—providing robust experimental and theoretical support for precision therapy of bone metastases and other solid tumors.

### 2.4 Evaluation of mitochondrial dysfunction and oxidative stress induced by ENCF

**Fig. 4:**
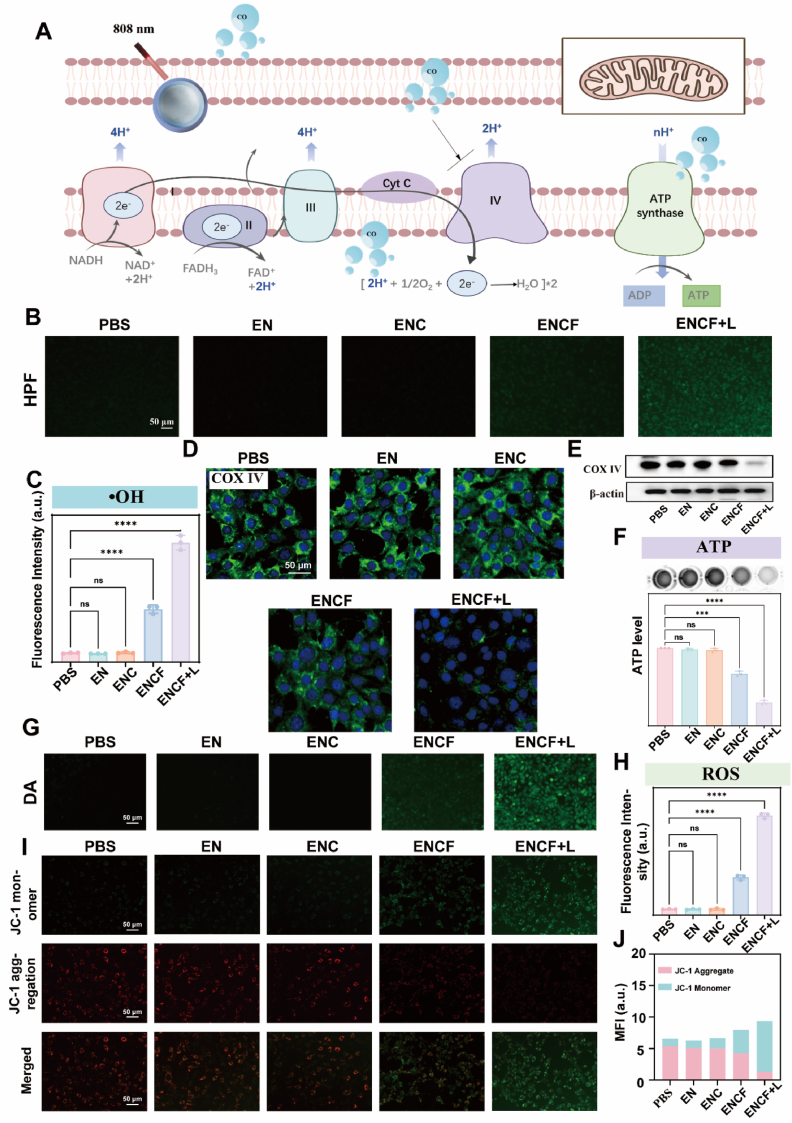
(A) Schematic illustration of the ENCF-triggered mitochondrial electron transport chain (ETC) blockade and ROS amplification under NIR light; (B) Fluorescence images of intracellular •OH levels detected by HPF probe under different treatments (scale bar: 50 μm); (C) Quantification of HPF fluorescence intensity across groups; (D) Immunofluorescence staining of COX IV expression after various treatments (scale bar: 50 μm); (E) Western blot analysis of COX IV protein levels; (F) Relative intracellular ATP levels measured via colorimetric assay; (G) Fluorescence images of total intracellular ROS visualized by DCFH-DA probe (scale bar: 50 μm); (H) Quantitative analysis of ROS fluorescence intensity normalized to PBS control; (I) JC-1 staining of mitochondrial membrane potential (ΔΨm) after different treatments (scale bar: 50 μm); (J) Quantification of red/green fluorescence ratio in JC-1 assay. All data are presented as mean ± SD (n = 3). Statistical analysis: ∗p < 0.05, ∗∗p < 0.01, ∗∗∗p < 0.001, n.s. = not significant.

To elucidate the molecular basis and underlying mechanisms by which the ENCF nanoplatform induces tumor cell death, this study focused on three major intracellular death-driving factors: mitochondrial dysfunction, reactive oxygen species (ROS) accumulation and energy metabolism disorders. As a highly regulated process, cell death typically involves crosstalk among multiple signaling pathways, with mitochondria serving as a central hub for energy metabolism and cell fate determination. Given their pivotal role in integrating oxidative stress signals, regulating ATP supply and initiating apoptosis or ferroptosis, mitochondria were selected as a critical focus in our investigation. Under NIR light activation, the C_3_N_4_-Fe shell of ENCF catalyzes the photoconversion of CO_2_ into CO via the upconverted emission from the EN core. The generated CO perturbs mitochondrial electron transport chain (ETC) function by competitively binding to the heme a_3_ site in cytochrome *c* oxidase (COX IV, Complex IV), thereby inhibiting oxygen's role as the terminal electron acceptor. This competitive inhibition disrupts electron flow and reduces proton translocation across the mitochondrial membrane, ultimately impairing the proton gradient and ATP synthase function (Fig. 4A). As a result, oxidative phosphorylation (OXPHOS) efficiency declines, compromising cellular energy supply and laying the foundation for metabolic imbalance and death signal activation. In addition to impairing OXPHOS, CO-mediated ETC disruption leads to electron leakage at Complexes I and III, promoting excessive superoxide anions (O_2_•^-^) [53] generation. These radicals subsequently react with Fe^2+^ to produce highly cytotoxic hydroxyl radicals (•OH) via Fenton-like reactions, further amplifying oxidative stress. To validate this mechanism, hydroxyphenyl fluorescein (HPF) was employed to detect intracellular •OH (Fig. 4B and C). Minimal fluorescence was observed in EN and ENC groups, whereas the ENCF group exhibited a moderate signal even without irradiation, indicating baseline ROS generation.

Upon NIR activation the ENCF + L group showed a 13-fold increase in fluorescence intensity relative to the EN group, confirming robust light-triggered •OH production and oxidative damage. To further confirm CO's inhibitory effect on COX IV, immunofluorescence and Western blot assays were performed (Fig. 4D and E). Both approaches revealed significantly reduced COX IV expression in the ENCF + L group, demonstrating that CO effectively blocks electron transfer, thereby inducing mitochondrial dysfunction and bioenergetic collapse. Consistent with this, ATP quantification (Fig. 4F) showed stable levels in PBS, EN and ENC groups, a moderate decrease in the ENCF group (66 % of PBS), and a dramatic reduction in the ENCF + L group (34 % of PBS), indicating severe impairment of energy metabolism and activation of cell death pathways. Overall ROS levels were quantified using DCFH-DA staining (Fig. 4G and H). The ENCF group exhibited ROS levels sevenfold higher than the PBS group, while ENCF + L reached an 18-fold increase. Combined with HPF-based detection of •OH, this result verifies that ROS “burst” arises from the synergistic effect of Fe-catalyzed reactions and ETC blockade, shifting the cell from redox homeostasis to an oxidative crisis that leads to irreversible damage. Mitochondrial membrane potential (MMP) was further evaluated using JC-1 staining (Fig. 4I and J). The PBS group exhibited a red/green fluorescence ratio of 4.42, indicative of intact MMP. By contrast, treatment with ENCF + L reduced this ratio to 0.16, signifying complete MMP collapse—a hallmark of irreversible cell death. This loss of MMP aligned closely with ATP depletion, confirming profound mitochondrial dysfunction. Collectively, these data establish a stepwise mechanism from structural damage, signal disruption and energetic exhaustion to ROS overproduction. The ENCF + L treatment effectively inhibits mitochondrial electron transport by downregulating COX IV, dramatically reduces ATP production and simultaneously promotes ROS accumulation via Fe-catalysis. This dual assault “energy starvation + oxidative stress” rapidly disrupts tumor cell homeostasis, leading to irreversible damage. Notably, MMP collapse serves as a “pre-mortem” marker within this cascade, underscoring mitochondria as the central therapeutic target of the ENCF-mediated photodynamic strategy. These findings not only provide mechanistic clarity but also establish a strong theoretical foundation for future in vivo validation and clinical translation.

### 2.5 ENCF triggers ferroptosis via ferritinophagy activation and antioxidant collapse

**Fig. 5:**
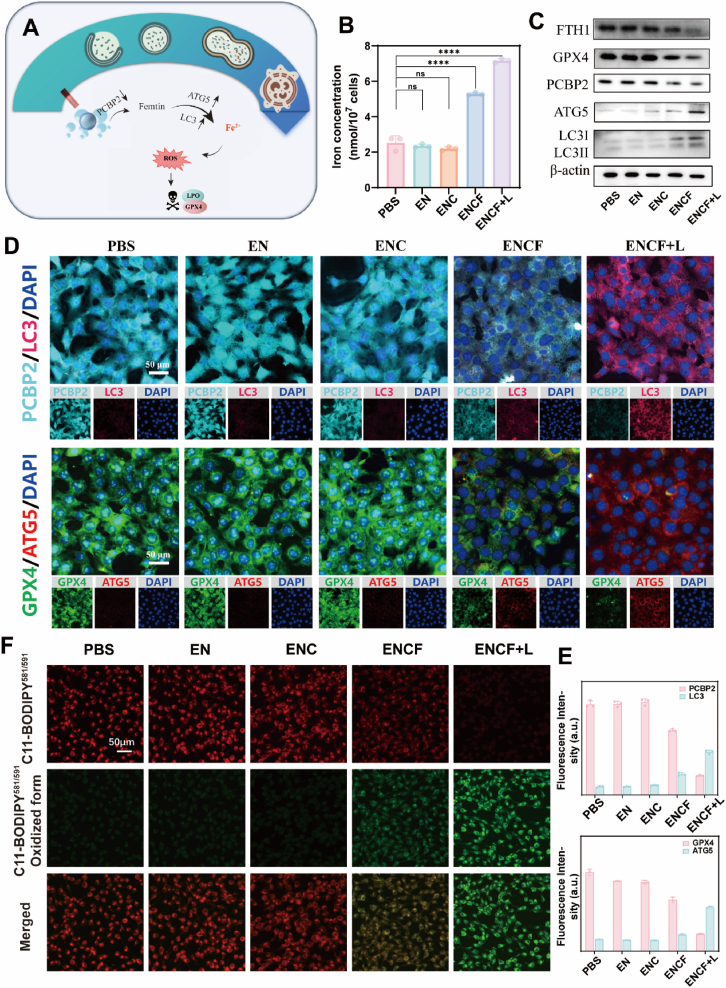
(A) Schematic of ferroptosis mechanism initiated by ENCF + L treatment; (B) Quantification of intracellular free Fe^2+^ levels in different treatment groups (n = 3); (C) Western blot analysis of ferroptosis (GPX4) and autophagy (PCBP2, ATG5, LC3 and FTH1) associated proteins. (D) Fluorescence images showing immunofluorescence staining of PCBP2 (Cyan)/LC3 (pink) and GPX4 (green)/ATG5 (red), scale bar: 50 μm; (E) Quantitative analysis of fluorescence intensity and co-localization in immunostained cells; (F) Lipid peroxidation detected by C11-BODIPY^581^/^591^; green fluorescence indicates oxidized lipids, red indicates non-oxidized (scale bar: 50 μm). (For interpretation of the references to color in this figure legend, the reader is referred to the Web version of this article.)

To further delineate the molecular mechanisms by which ENCF induces tumor cell death, we investigated ferroptosis, a non-classical, iron-dependent form of programmed cell death distinct from apoptosis and necrosis. Ferroptosis is driven by Fe^2+^-catalyzed lipid peroxidation and the collapse of antioxidant defenses, most notably glutathione peroxidase 4 (GPX4) [54,55]. Mechanistically, Fe^2+^ promotes the Fenton reaction (Fe^2+^ + H_2_O_2_ → Fe^3+^ + •OH + OH^−^), generating highly reactive hydroxyl radicals (•OH) that attack phospholipids enriched in polyunsaturated fatty acids (PUFAs). This results in the accumulation of lipid peroxides (L-OOH), which, if not detoxified by GPX4, cause irreversible membrane damage and cell death. To assess whether ENCF disrupts intracellular iron homeostasis, we quantified free Fe^2+^ levels using a colorimetric assay (Fig. 5B). In PBS, EN and ENC groups, free Fe^2+^ levels remained stable (2.52, 2.35 and 2.19 nmol/10^7^ cells, respectively). In the ENCF group without light, a moderate elevation to 5.27 nmol/10^7^ cells was observed, still within the regulatory capacity of iron homeostasis. Notably, the ENCF + L group showed a dramatic increase to 7.16 nmol/10^7^ cells (2.84-fold higher than PBS), suggesting light-triggered iron overload due to FTH1 disassembly, impaired storage and disturbed transport—key events initiating ferroptotic cascades. To further elucidate the source of Fe^2+^ in the Fenton reaction, we performed in vitro degradation experiments under acidic pH with H_2_O_2_. ENCF showed negligible Fe^2+^ release at pH 7.0–4.0, even in the presence of H_2_O_2_, indicating high structural stability under physiologically relevant conditions. Appreciable Fe^2+^ release was only observed at pH ≤ 3.0, which is beyond the tumor microenvironment range. The colorimetric assay yielded a green signal, attributable to the overlap of the intrinsic yellow color of ENCF and K_3_[Fe(CN)_6_] with the characteristic blue of Prussian Blue (Fig. S5). These results confirm that under realistic conditions, Fe^2+^ mainly originates from intracellular autophagy, with ENCF acting as a catalytic platform rather than a direct Fe^2+^ reservoir. Western blotting further revealed a marked reduction in FTH1 expression to control levels, directly confirming FTH1 degradation and enhanced Fe^2+^ release. We next examined key regulatory proteins by Western blot and immunofluorescence (Fig. 5C and D). PCBP2, a cytosolic iron chaperone essential for Fe^2+^ trafficking, was markedly downregulated by 79 % in the ENCF + L group, consistent with ROS-mediated disruption of iron transport [56]. Concurrently, expression of autophagy-related protein ATG5 increased 2.74-fold, and LC3 levels increased from 7.22 to 37.77, indicating significant activation of ferritinophagy. In parallel, GPX4 protein levels plummeted to below 25 % of control, indicating collapse of the antioxidant system. Fluorescence microscopy further corroborated these events, showing cytoplasmic colocalization of PCBP2 (cyan) and LC3 (pink) in ENCF + L, consistent with impaired iron transport and autophagic engagement. ATG5 (red) was strongly upregulated, while GPX4 (green) signals declined markedly. To determine whether lipid peroxidation progressed to terminal execution stages, we employed the oxidation-sensitive probe C11-BODIPY^581^/^591^ (Fig. 5F). Upon oxidation, red fluorescence shifts to green. In the ENCF + L group, green fluorescence intensity increased 3.33-fold over control, while red signal declined sharply, yielding a green/red ratio of 4.2 (Fig. S6). This indicates extreme lipid oxidative stress and catastrophic membrane oxidative damage. Together, these results confirm that ENCF + L induces ferroptosis through a multi-step cascade: (i) PCBP2 downregulation disrupts Fe^2+^ trafficking, leading to cytosolic accumulation; (ii) ferritinophagy activation via ATG5 and LC3 promotes FTH1 degradation and endogenous Fe^2+^ release; and (iii) GPX4 downregulation abrogates lipid peroxide detoxification, driving irreversible oxidative collapse (Fig. 5A). This integrated ferroptotic network, iron dysregulation, autophagic disinhibition, and antioxidant breakdown, establishes the mechanistic foundation for ENCF-mediated tumor killing and highlights its translational potential for ferroptosis-based cancer therapy.

### 2.6 In vivo NIR-II fluorescence imaging and therapeutic evaluation of ENCF in bone metastasis mouse model

**Fig. 6:**
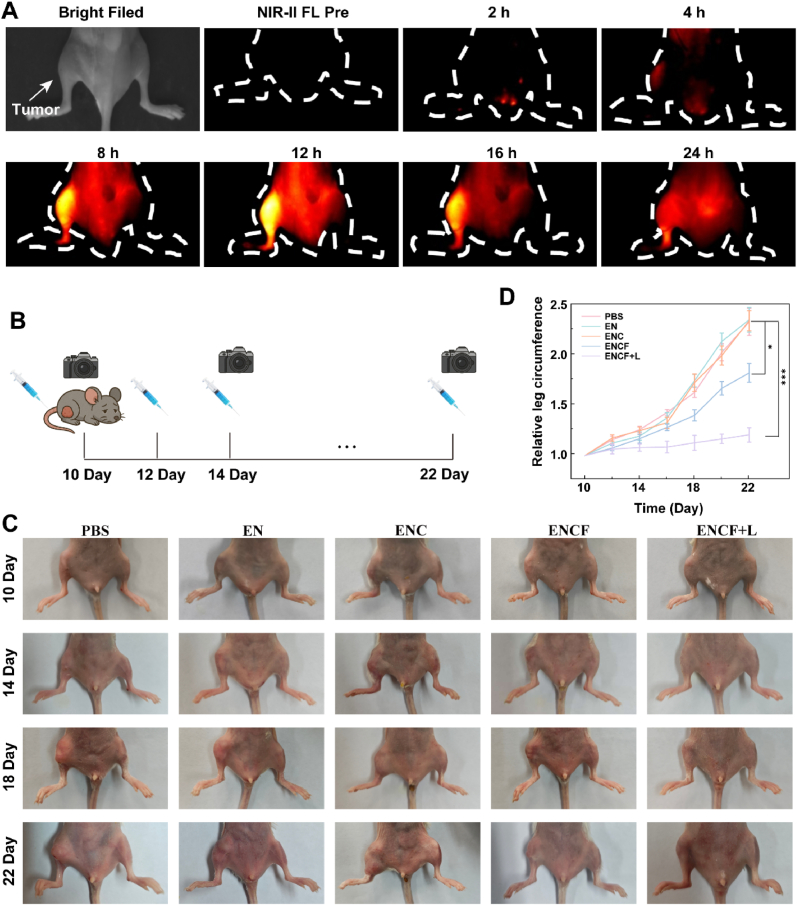
(A) Time-resolved NIR-II fluorescence imaging of mice after intravenous injection of ENCF (5 mg/mL, 100 μL). The arrow indicates the tumor site; (B) Schematic diagram of experimental timeline and treatment schedule in the bone metastasis model; (C) Representative photographs of 4T1 bone metastasis model mice under different treatment groups; (D) Relative leg circumference curves of bone metastasis mice in different groups over 12 days (n = 5). Data are shown as mean ± SD; statistical significance assessed via two-way ANOVA: ∗p < 0.05, ∗∗p < 0.01, ∗∗∗p < 0.001.

To systematically evaluate the targeting accumulation capability and visual imaging performance of the ENCF nanoplatform in the complex in vivo tumor microenvironment, an orthotopic bone metastasis mouse model was first established. 4T1 breast cancer cells were slowly injected into the right tibial bone marrow cavity of Balb/c mice to generate localized bone-destructive lesions. This model closely mimics clinical bone metastases from breast cancer in terms of tissue invasion patterns, metastatic tropism and bone degradation morphology, providing high reproducibility and experimental controllability. To rigorously confirm the successful establishment of the bone metastasis model, we conducted X-ray radiography and Hematoxylin and eosin (H&E) staining on the tumor-bearing tibia. As illustrated in Fig. S7, X-ray imaging clearly revealed progressive osteolytic lesions characterized by cortical bone discontinuity, trabecular rarefaction, and marked reduction in bone density in the tumor-bearing tibia, while the contralateral tibia remained intact. Correspondingly, H&E staining (Fig. S8) showed massive infiltration of malignant tumor cells within the bone marrow cavity, accompanied by destruction of the trabecular bone and cortical thinning. Moreover, tumor cells were observed breaching the cortical bone and extending into surrounding soft tissues, which is pathognomonic for advanced bone metastasis. Following successful model establishment, mice received intravenous injection of ENCF (100 μL, 5 mg/mL). NIR-II (1000–1700 nm) fluorescence imaging was then performed at multiple time points post-injection to dynamically monitor the biodistribution and accumulation of ENCF in vivo. As shown in Fig. 6A, the first panel depicts the bright-field image of the mouse, with arrows indicating the tumor-bearing tibial side, and the second panel shows the baseline NIR-II fluorescence image acquired prior to ENCF administration. The subsequent panels present time-dependent NIR-II fluorescence signals following probe injection. Thanks to the deep-tissue penetration, low background interference and reduced scattering of NIR-II imaging, strong fluorescence signals in the bone metastasis region emerged as early as 4 h post-injection, reaching peak intensity at 12 h, and maintaining a high-level signal plateau between 8 and 16 h. Notably, fluorescence intensities at the metastatic site were significantly higher than those in the surrounding tissues, indicating effective tumor-targeting accumulation. This preferential enrichment of ENCF is primarily attributed to the enhanced permeability and retention (EPR) effect in the bone tumor microenvironment, which allows nanoparticles of optimal size and surface properties to extravasate from the vasculature and be retained within tumor tissues. This passive targeting mechanism ensures efficient accumulation of ENCF at the metastatic site, providing a solid foundation for subsequent therapeutic applications. To further validate the fluorescence imaging findings, quantitative biodistribution analysis was conducted using inductively coupled plasma (ICP). After subtraction of the basal Fe content from healthy mice, the Fe concentration derived from ENCF was quantified in major organs and tumor-bearing bone at multiple time points post-injection (Fig. S9). The results demonstrated that ENCF rapidly entered the liver from the bloodstream, showing the highest hepatic concentration within the first 1 h, followed by a gradual decline. Consistent with the NIR-II imaging results, the Fe content in the bone metastatic site peaked at 12 h post-injection, coinciding with the strongest fluorescence signal. At 24 h, liver Fe levels dropped markedly, indicating effective systemic clearance. Pharmacokinetic analysis revealed that ENCF was predominantly metabolized via hepatobiliary pathways. These findings not only corroborate the NIR-II imaging results but also confirm the favorable metabolic clearance profile of the nanoplatform. Collectively, the combination of X-ray/HE pathological validation, NIR-II dynamic imaging and ICP biodistribution analysis, not only demonstrates that the ENCF nanoplatform preferentially accumulates in bone metastases but also confirms the robust establishment of the bone metastasis model, thereby providing a reliable foundation for evaluating therapeutic efficacy. These results strongly support the potential of the ENCF system for image-guided, tumor-targeted, and phototriggered therapy, offering a promising and precise strategy for effective treatment of metastatic bone tumor.

Subsequently, to evaluate in vivo therapeutic efficacy, tumor-bearing mice were randomly divided into five groups (n = 5): PBS, EN, ENC, ENCF and ENCF + L groups. In the ENCF + L group, mice received 808 nm laser irradiation (1 W/cm^2^, 10 min) at 8 h post-injection, coinciding with peak tumor accumulation. Treatments were repeated every two days. Tumor progression was tracked by measuring leg circumference bi-daily and photographing lesion sites every four days (Fig. 6B). As shown in Fig. 6C and D, PBS, EN and ENC groups exhibited rapid tumor growth, with final leg circumference increasing to approximately 2.3-fold of baseline. In contrast, the ENCF group without laser activation already demonstrated moderate tumor growth inhibition, indicating the intrinsic therapeutic potential of the nanoplatform. Notably, the ENCF + L group showed the most significant anti-tumor effect: tumor progression was markedly suppressed starting from day 18, with average leg circumference reaching only 1.2-fold of baseline by day 22, highlighting the powerful therapeutic synergy between the nanoplatform and laser-triggered activation. Importantly, no significant body weight changes were observed across all groups during treatment, further supporting the favorable biosafety profile of ENCF (Fig. S10).

### 2.7 Evaluation of bone structural preservation of ENCF nanoplatform

**Fig. 7:**
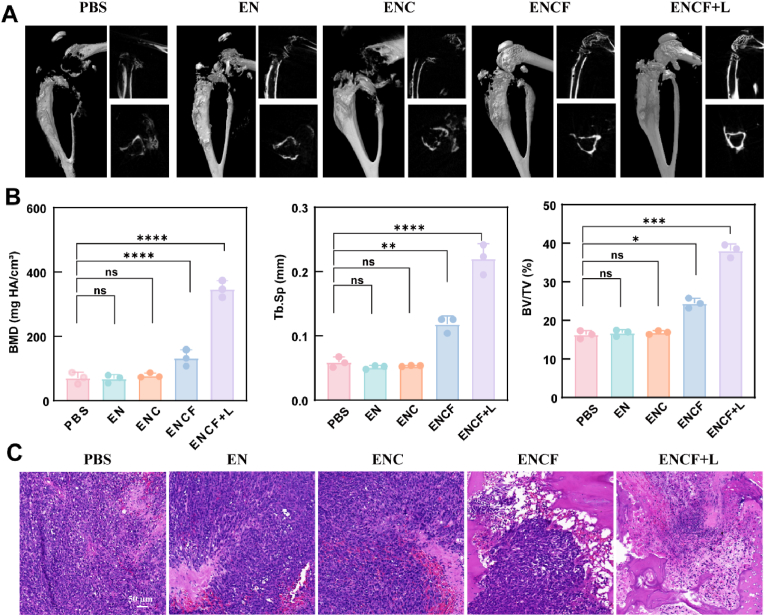
(A) Representative micro-CT images of tibial metastatic lesions after treatment. (B) Quantitative analysis of bone parameters: Bone mineral density (BMD), trabecular separation (Tb.Sp) and bone volume fraction (BV/TV). (C) Histological evaluation of decalcified bone sections using H&E. Data are expressed as mean ± SD (n = 5); statistical analysis via one-way ANOVA: ∗p < 0.05, ∗∗p < 0.01, ∗∗∗p < 0.001.

To further validate the bone-protective efficacy of ENCF in a bone metastasis model, we systematically assessed its impact on bone structural integrity. High-resolution micro-computed tomography (micro-CT) was employed to visualize and quantify tumor-induced osteolytic destruction and skeletal changes in the tibiae of mice from different treatment groups. Prior to scanning, the micro-CT system was carefully calibrated using a hydroxyapatite phantom to ensure quantitative accuracy of bone mineral density and morphometric parameters. As shown in the reconstructed 3D micro-CT images (Fig. 7A), mice in the PBS, EN and ENC groups exhibited severe bone destruction, including marked deformity of the medullary cavity, extensive trabecular loss, cortical thinning, and in some cases cortical fractures with blurred boundaries. In contrast, mice in the ENCF group displayed modest attenuation of bone destruction, with partial preservation of cortical structures, suggesting a moderate protective effect. Remarkably, the ENCF + L group exhibited the most prominent protective phenotype, retaining relatively intact cortical boundaries and preserved skeletal architecture, thereby demonstrating that laser-activated ENCF treatment can effectively suppress tumor-driven osteolysis. Quantitative micro-CT data (Fig. 7B) further confirmed these imaging observations. The calibration-corrected measurements confirmed that bone mineral density (BMD) in the ENCF + L group reached 347.44 mg HA/cm^3^, significantly higher than the PBS (70.76 mg HA/cm^3^) and ENCF (132.89 mg HA/cm^3^) groups, suggesting strong attenuation of tumor-driven bone loss. Similarly, the trabecular separation (Tb.Sp), an index of trabecular spacing, was lowest in the PBS group (0.059 mm) and greatest in the ENCF + L group (0.22 mm), indicating the preservation of a relative compact trabecular network. In terms of bone volume fraction (BV/TV), the ENCF + L group reached 38 %, which was substantially higher than that of the PBS (16 %) and ENCF (24 %) groups, demonstrating significant retention of bone volume and structural integrity. Histological evaluation via H&E staining (Fig. 7C) further confirmed the protective effect at the tissue level. Extensive tumor infiltration and bone resorption were observed in the PBS group, with no significant improvement in the EN or ENC groups. The ENCF group showed partial repair with persistent tumor presence. In contrast, the ENCF + L group exhibited greatly reduced tumor infiltration and preserved cortical bone integrity, indicating an effective dual impact on both tumor suppression and bone protection.

**Fig. 8:**
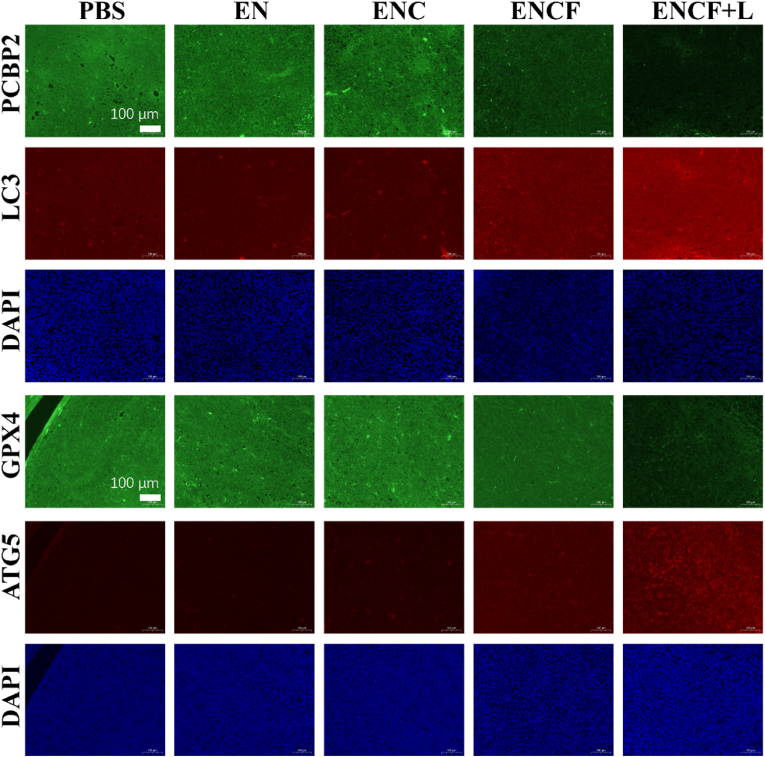
Immunofluorescence staining for PCBP2, LC3, GPX4 and ATG5.

To further validate whether the therapeutic mechanism of ENCF in vivo is consistent with the cellular findings, immunofluorescence staining of tumor tissues was performed for representative molecular markers, including GPX4, LC3, PCBP2, and ATG5. As shown in Fig. 8, GPX4 expression was markedly decreased in the ENCF + L group compared with control groups, consistent with activation of ferroptosis. LC3 and ATG5 signals were significantly enhanced, indicating robust induction of autophagic flux and autophagosome formation. Meanwhile, PCBP2 expression was reduced, reflecting impaired iron homeostasis and contributing to iron-driven oxidative stress. Taken together, the in vivo immunofluorescence data strongly support that ENCF exerts its anti-tumor effect through the same mechanistic pathway as identified in vitro. By integrating both in vitro and in vivo evidence, we demonstrate a coherent and reproducible mechanism, thereby reinforcing the reliability of our proposed therapeutic strategy.

Comprehensive in vivo biosafety assessment was carried out by examining systemic toxicity and hematological indicators at the end of treatment. H&E staining of major organs (heart, liver, spleen, lung, and kidney) showed no evident histopathological abnormalities, such as inflammatory infiltration, necrosis, or tissue damage, indicating good biocompatibility of ENCF (Fig. S11). In addition, serum biochemical markers including creatinine (CRE), alanine aminotransferase (ALT), blood urea nitrogen (BUN), and aspartate aminotransferase (AST) remained within normal ranges across groups, suggesting no significant hepatic or renal impairment (Fig. S12). Furthermore, hematological analysis revealed stable levels of red blood cells (RBC), white blood cells (WBC), and platelets (PLT), which indicates abcence of hematotoxicity or immunological disturbances (Fig. S13). In addition, hemolysis experiments showed negligible erythrocyte disruption across all tested concentrations of ENCF, indicating excellent hemocompatibility (Fig. S14). Collectively, these results demonstrate that ENCF exhibits favorable systemic safety and negligible off-target toxicity during therapeutic application.

In conclusion, the ENCF nanoplatform, which shows favorable biosafety, enables precise lesion localization via NIR-II fluorescence imaging and, upon laser activation, triggers a cascade of CO, Fe^2+^ and ROS-mediated ferroptosis and oxidative stress pathways, leading to effective inhibition of metastatic bone tumor progression. The anti-tumor effect in turn alleviates tumor-driven bone destruction, delays bone loss and highlights the platform's theranostic potential. This dual-functionality system offers a promising multimodal intervention strategy for deeply seated metastatic bone tumors.

## 3 Conclusions

In this study, we developed a NIR-II fluorescence-guided photocatalytic nanoplatform (ENCF) that integrates real-time imaging, tumor-specific CO release, Fenton-like hydroxyl radical generation and iron autophagy activation to synergistically induce ferroptosis for effective treatment of bone metastases. Upon 808 nm laser irradiation, ENCF enabled efficient photocatalytic release of CO, which disrupted mitochondrial electron transport, suppressed ATP synthesis and promoted endogenous ROS accumulation. Simultaneously, Fe within the platform catalyzed H_2_O_2_ into cytotoxic •OH through a Fenton-like reaction, amplifying oxidative stress. Mechanistically, ENCF further activated ferritinophagy by downregulating PCBP2 and upregulating LC3 and ATG5, leading to FTH1 degradation and intracellular Fe^2+^ release, which intensified lipid peroxidation and facilitated GPX4 depletion. These synergistic events collectively triggered robust ferroptotic cell death. In vivo studies using a bone metastasis model demonstrated that ENCF significantly suppressed tumor progression, reduced osteolytic damage and exhibited favorable biosafety. Overall, our results demonstrate that the ENCF platform provides a multifaceted and precisely controllable strategy for the treatment of deep-seated metastatic tumors, particularly by leveraging CO-enhanced ferritinophagy and ferroptosis under NIR-II photoactivation.

## CRediT authorship contribution statement

**Qin Liu:** Writing – original draft, Investigation, Funding acquisition, Formal analysis, Data curation. **Jian Zhang:** Visualization, Investigation, Conceptualization. **Lujie Yu:** Visualization, Validation, Methodology, Formal analysis, Data curation. **Yaohua Chen:** Visualization, Formal analysis, Data curation. **Chiyv Zhang:** Investigation, Formal analysis, Data curation. **Juan Li:** Visualization, Investigation. **Shutong Wu:** Visualization, Investigation. **Xiaochun Zheng:** Visualization, Investigation. **Rong Dai:** Validation, Supervision, Data curation. **Ziliang Zheng:** Writing – review & editing, Supervision, Methodology, Funding acquisition, Conceptualization. **Ruiping Zhang:** Supervision, Project administration, Funding acquisition, Conceptualization.

## Declaration of competing interest

The authors declare that they have no known competing financial interests or personal relationships that could have appeared to influence the work reported in this paper.

## Data Availability

Data will be made available on request.
