# Supplementary material for: Light-triggered carbon monoxide-induced activation of enhanced ferritinophagy-mediated ferroptosis for bone metastases therapy
Source: Mater Today Bio. 2025 Sep 17;35:102322. doi: 10.1016/j.mtbio.2025.102322 (PMC12513188; doi:10.1016/j.mtbio.2025.102322)
Supplement: Multimedia component 1 [file mmc1.docx]

Supporting Information

**Light-triggered carbon monoxide-induced activation of enhanced ferritinophagy-mediated ferroptosis for bone metastases therapy**

Experimental Section

1. Materials.

Neodymium(III) acetate hexahydrate (Nd(C₂H₃O₂)₃·6H₂O, 99.9%), Yttrium(III) acetate hexahydrate (Y(C₂H₃O₂)₃·6H₂O, 99.9%), Ytterbium(III) acetate hexahydrate (Yb(CH₃COO)₃·6H₂O, 99.9%), and Erbium(III) acetate (Er(C₂H₃O₂)₃·xH₂O, 99.9%) were acquired from Aladdin Industrial Corporation (Shanghai, China). NH₄F, oleic acid (OA), 1-octadecene (ODE), urea, and citric acid were also obtained from the same supplier. Dulbecco’s Modified Eagle Medium (DMEM) and fetal bovine serum (FBS) were sourced from Gibco BRL (Grand Island, NY, USA). Cell Counting Kit-8 (CCK-8) and 2′,7′-dichlorodihydrofluorescein diacetate (DCFH-DA) were provided by Sigma-Aldrich (Shanghai) Trading Co., Ltd. Ultrapure deionized water was used in all experiments. Unless otherwise specified, all reagents were used as received without further purification.

2. Characterization.

A JEOL-2100F transmission electron microscope (TEM) was employed to observe the morphology and dispersion of the nanoparticles. X-ray photoelectron spectroscopy (XPS) was performed on a Thermo Fisher ESCALAB 250Xi spectrometer to analyze the surface chemical composition. Fourier transform infrared (FT-IR) spectra were collected using a Vertex PerkinElmer 580BIR spectrometer (Bruker) via the KBr pellet method to identify chemical bonds and estimate their relative abundance. Dynamic light scattering (DLS) and zeta potential measurements were carried out with a Nano-Zetasizer (Malvern Instruments Ltd, UK). Photoluminescence spectra were acquired using an FLS980 spectrometer (Edinburgh Instruments, UK). UV–vis absorption spectra were obtained using a TU-1901 spectrophotometer (PerkinElmer), and near-infrared-II (NIR-II) fluorescence emission spectra were measured using a system provided by NIR-Optics Co., Ltd. (Suzhou, China) to assess optical properties.

3. Synthesis of β-NaYF_4_:18%Yb, 2%Er (E) Cores

NaYF₄:18%Yb,2%Er nanoparticles were synthesized via a thermal co-precipitation method. In a 100 mL three-necked flask, 0.8 mmol Y(CH₃COO)₃, 0.18 mmol Yb(CH₃COO)₃, and 0.02 mmol Er(CH₃COO)₃ were mixed with 3 mL oleic acid (OA) and 17 mL 1-octadecene (ODE). The mixture was heated to 150 °C and maintained for 1 hour under magnetic stirring to ensure complete ligand coordination. After cooling to room temperature, 8 mL of methanol solution containing 0.1 g NaOH and 0.148 g NH₄F was slowly added dropwise. The reaction mixture was stirred for another hour and then gradually heated to 90 °C for 30 min under reduced pressure to remove excess methanol. Subsequently, the temperature was raised to 300 °C under a nitrogen atmosphere and maintained for 1 hour to complete crystal growth. After naturally cooling to room temperature, the resulting product was collected by centrifugation at 8,000 rpm for 10 minutes, washed twice with hexane and ethanol, and centrifuged again. The final precipitate was redispersed in 8 mL hexane, followed by centrifugation at 8,000 rpm. The supernatant was retained for future use.

4. Synthesis of NaYF_4_:18%Yb, 2%Er@NaYF_4_:20%Nd (EN) Nanoparticles

Core–shell NaYF₄-based nanoparticles were synthesized via a controlled epitaxial growth method. Briefly, 0.8 mmol Y(CH₃COO)₃ and 0.2 mmol Nd(CH₃COO)₃ were dissolved in a mixture of 3 mL oleic acid (OA) and 17 mL 1-octadecene (ODE) in a 100 mL three-neck flask. The solution was heated to 150 °C and maintained for approximately 1 hour under continuous stirring until a clear, optically transparent solution was obtained, indicating complete complexation of lanthanide precursors. After cooling to room temperature, 8 mL of methanol containing 0.1 g NaOH and 0.148 g NH₄F was slowly added to the flask, followed by the dropwise addition of 4 mL of hexane dispersion containing the pre-synthesized NaYF₄:18%Yb,2%Er core nanoparticles. The resulting mixture was stirred for 1 hour to facilitate surface adsorption, then gradually heated to 90 °C to evaporate residual volatile solvents. Subsequently, the temperature was raised to 300 °C under a nitrogen atmosphere and maintained for 1 hour to promote uniform shell growth. After natural cooling to room temperature, excess hexane and ethanol were added to the mixture to precipitate the core–shell nanoparticles. The product was collected by centrifugation and washed twice with hexane and ethanol to remove unreacted species. Finally, the purified nanocrystals were redispersed in cyclohexane for storage and further use.

5. Synthesis of ENC and ENCF nanoparticles

Typically, 1 mL of oleic acid-capped EN (10 mg/mL in cyclohexane) is mixed with 3 mL cyclohexane, 3 mL N,N-dimethylformamide (DMF), and NOBF₄ (5.8 mg, 50 μmol) in a centrifuge tube. The mixture is vigorously vortexed at room temperature for 10 minutes and then allowed to stand for 5 minutes to facilitate phase transfer. The upper cyclohexane layer is then discarded, and 3 mL of isopropanol is added to precipitate the nanoparticles. The modified EN are collected by centrifugation and redispersed in 0.5 mL of DMF. This DMF dispersion is mixed with citric acid and stirred vigorously for 2 hours. After thorough washing with water using ultrafiltration tubes to remove residual reagents, the product is dried in a vacuum oven. The dried powder is then thoroughly ground with citric acid, urea, and ferrous sulfate, and transferred to a reaction vessel for thermal treatment at 180 °C for 3 hours. Upon completion of the reaction, the product is washed repeatedly with water to remove unbound C_3_N_4_, yielding the final ENCF nanoparticles.

6. Measurement of CO Release from ENCF via Hemoglobin Assay

Carbon monoxide (CO) release from ENCF under Laser was evaluated by monitoring the spectral conversion of deoxyhemoglobin (Hb) to carboxyhemoglobin (HbCO) based on their characteristic absorption peaks. Bovine hemoglobin (MP Biomedicals) was initially dissolved in phosphate-buffered saline (PBS) to a final concentration of 4.2 μM. The solution was then deoxygenated by reduction with sodium dithionite under a nitrogen atmosphere to yield deoxyhemoglobin. Subsequently, ENCF was also deoxygenated under nitrogen bubbling and added to the deoxygenated hemoglobin solution. The resulting mixture was transferred to a UV-grade quartz cuvette and incubated under nitrogen atmosphere. UV-vis absorption spectra were recorded at different time intervals over a wavelength range of 350 to 600 nm to monitor the conversion process. To ensure accurate quantification and minimize spectral interference, the absorbance changes at 410 nm and 430 nm—representing the characteristic peaks of Hb and HbCO, respectively—were utilized. The percentage of Hb converted to HbCO was calculated using the following equation:

C_CO_=$\frac{\text{528.6*}\text{I}_{\text{410 nm}}\text{-304*}\text{I}_{\text{430 nm}}}{\text{216.5*}\text{I}_{\text{410 nm}}\text{+442.4*}\text{I}_{\text{430 nm}}}$*C_Hb_

7. Measurement of CO Release from ENCF via GC

For the photocatalytic CO release experiments, 5 mg of ENCF powder was dispersed in 5 mL of deionized water to obtain a stable suspension. Prior to laser irradiation, CO₂ was continuously bubbled through the suspension at a constant flow rate of 0.5 mL·min⁻¹ for 30 min, which effectively removed residual air and ensured dissolution equilibrium. The system was then irradiated with an 808 nm laser (1 W·cm⁻^2^) under continuous CO_2_ flow, during which the gaseous products evolved from the reaction were collected online. These gases were directly introduced into an Agilent GC-7890 gas chromatograph equipped with a thermal conductivity detector, and the amount of CO produced was quantitatively determined by comparing the retention times and peak areas with those of authentic CO standards.

8. Cell culture

4T1 cells are cultured in DMEM cell culture medium supplemented with 10% fetal bovine serum (FBS) and 1% penicillin-streptomycin solution. The culture is maintained in an incubator at 37°C with a 5% CO₂ atmosphere. Cells should be regularly monitored for confluency, and passaged when they reach 70-80% confluence to ensure healthy growth and prevent overcrowding. During passaging, cells are washed with phosphate-buffered saline (PBS) and detached using trypsin-EDTA solution. After neutralizing trypsin with fresh culture medium, cells are centrifuged, resuspended in fresh culture medium, and seeded into new culture vessels at appropriate densities.

9. Cellular uptake

For the cell uptake experiment, 4T1 cells were seeded in 48-well plates at a density of 2×10^4^ cells and cultured for 24 hours, followed by co-incubation with 500 µg/mL ENCF for different time intervals (0, 0.5, 1 and 2 hours). Subsequently, the fluorescence intensity of ENCF in cells was evaluated using near-infrared II (NIR-II) fluorescence microscopy.

10. Cell viability

4T1 cells were seeded into 96-well plates at a density of 1×10⁴ cells per well and cultured for 24 hours. Then the cells were treated with various solutions for another 24 hours, including: (a) PBS; (b) EN; (c) ENC; (d) ENCF; and (e) ENCF+L (with 808 nm laser irradiation). Cell viability was evaluated using a CCK-8 assay kit. Specifically, 10 μL of CCK-8 solution was added to each well of the 96-well plate. After 1 hour of incubation, the absorbance of cells at 450 nm was measured using a microplate reader.

11. Western Blotting Assay

For protein expression analysis, 4T1 cells cultured on the corresponding samples were harvested and lysed on ice for 30 minutes using RIPA lysis buffer supplemented with protease inhibitors. The lysates were centrifuged at 12,000 rpm for 15 minutes, and the supernatants (total protein) were collected. Protein concentrations were determined using the BCA assay, and equal amounts of protein were mixed with loading buffer and boiled for 5 minutes for denaturation. Subsequently, the protein samples were separated via SDS-PAGE and transferred onto PVDF membranes using the wet transfer method. After transfer, membranes were blocked with 5% non-fat milk for 1 hour at room temperature, followed by washing with TBST. The membranes were then incubated overnight at 4 °C with primary antibodies (COX IV, GPX4, PCBP2, ATG5, LC3, and β-actin). After washing, the membranes were incubated with HRP-conjugated secondary antibodies (1:5000) for 1 hour at room temperature. Protein bands were visualized using a chemiluminescence imaging system, and band intensities were quantified using ImageJ software. β-actin was used as the internal loading control to normalize the relative expression levels of the target proteins.

12. Immunofluorescent Staining

Cells were fixed with 4% paraformaldehyde, permeabilized with 0.5% Triton X-100, and blocked with 5% BSA. Subsequently, cells were incubated with the primary antibodies overnight at 4 °C, followed by incubation with Alexa Fluor 488-conjugated secondary antibodies at 37 °C for 1 hour. Nuclei were counterstained with DAPI (1:1000 dilution), and fluorescent images were captured using an inverted fluorescence microscope.

13. Animal Model

All animal experiments were conducted in accordance with protocols approved by the Institutional Animal Care and Use Committee (IACUC) of the Animal Experiment Center, Shanxi Medical University. Female BALB/c mice (6–8 weeks old) were obtained from Charles River Laboratory Animal Technology Co., Ltd. (Beijing, China). To establish an orthotopic bone tumor model, mice were anesthetized using isoflurane and maintained under anesthesia throughout the procedure. Subsequently, 1 × 10⁶ 4T1 cells suspended in 50 μL of phosphate-buffered saline (PBS) were subcutaneously injected into the right thigh of each mouse. During all procedures, body temperature was monitored and maintained using a temperature-controlled heating device to ensure animal welfare.

14. In Vivo Fluorescence Imaging

To assess the biodistribution of ENCF nanoparticles, in vivo NIR-II fluorescence imaging was performed in tumor-bearing mice following intravenous administration of ENCF at a dose of 20 mg kg⁻¹. Whole-body fluorescence images were captured at predetermined time intervals (0, 2, 4, 6, 8, 10, 12, and 24 hours post-injection) using a small animal imaging system (NIR-Optics Co., Ltd., Suzhou, China) equipped with an II900/1700 series detector suitable for NIR-II signal acquisition.

15. Statistical Analysis

All statistical analyses were performed using Origin 2018 and ImageJ software. Data are presented as mean ± standard deviation (SD) from at least three independent experiments. One-way two-tailed analysis of variance (ANOVA) followed by Tukey’s post hoc multiple comparisons test was used to assess statistical significance among groups. A *p* value less than 0.05 was considered statistically significant (*p* < 0.05, *p* < 0.01, *p* < 0.001). No data transformations, normalization procedures, or outlier exclusions were applied in this study.


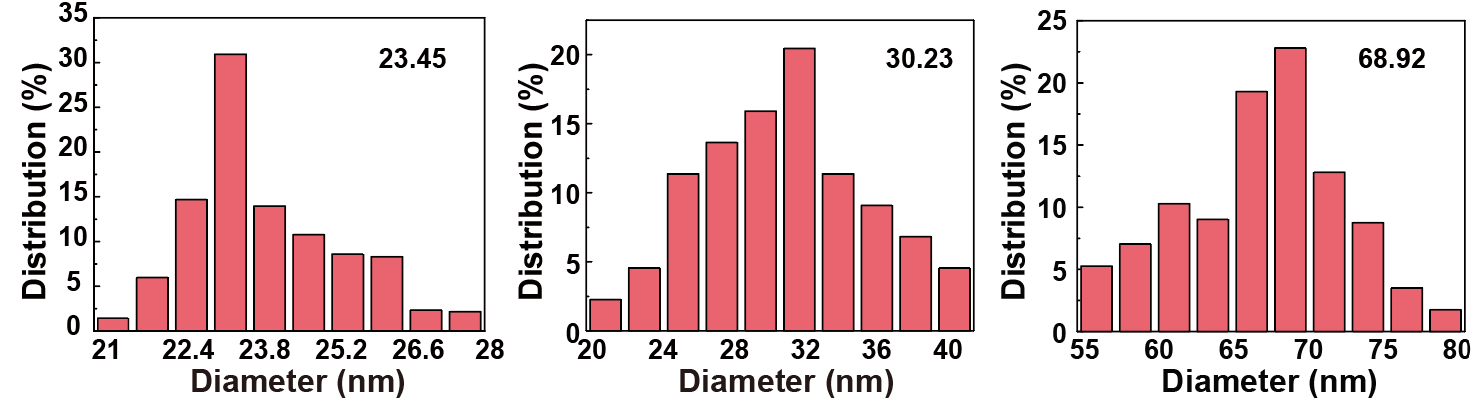


**Figure S1.** Particle size distribution of E, EN and ENCF.


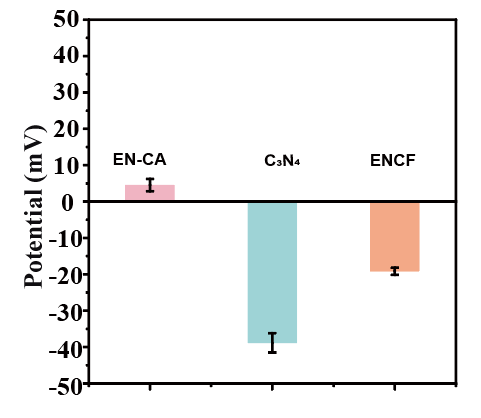


**Figure S2.** Zeta potential of EN-CA, C_3_N_4_ and ENCF.


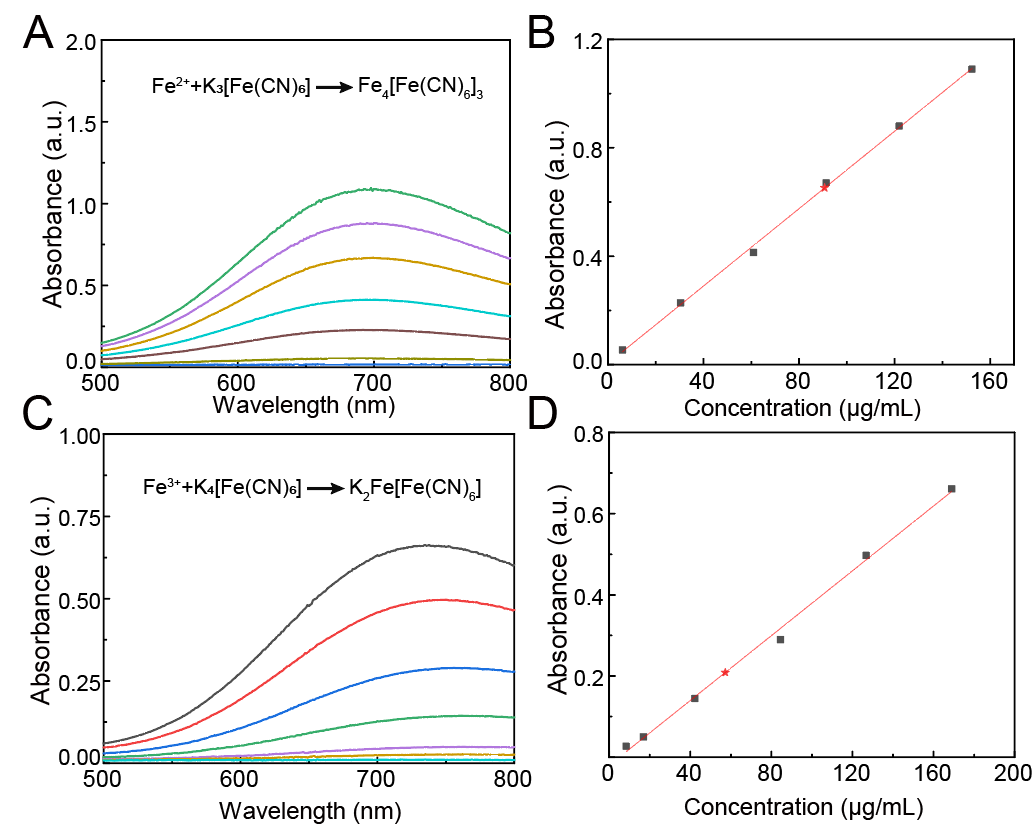


**Figure S3.** Standard calibration curves for Fe^2^⁺ and Fe^3^⁺ using potassium ferrocyanide and potassium ferricyanide, respectively, and quantitative determination of Fe^2^⁺/Fe^3^⁺ released from ENCF.


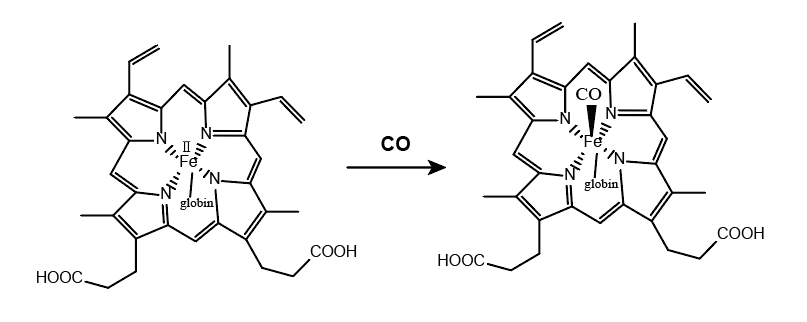


**Figure S4.** Schematic of the hemoglobin (Hb)-based CO detection method using absorbance at 420  nm.


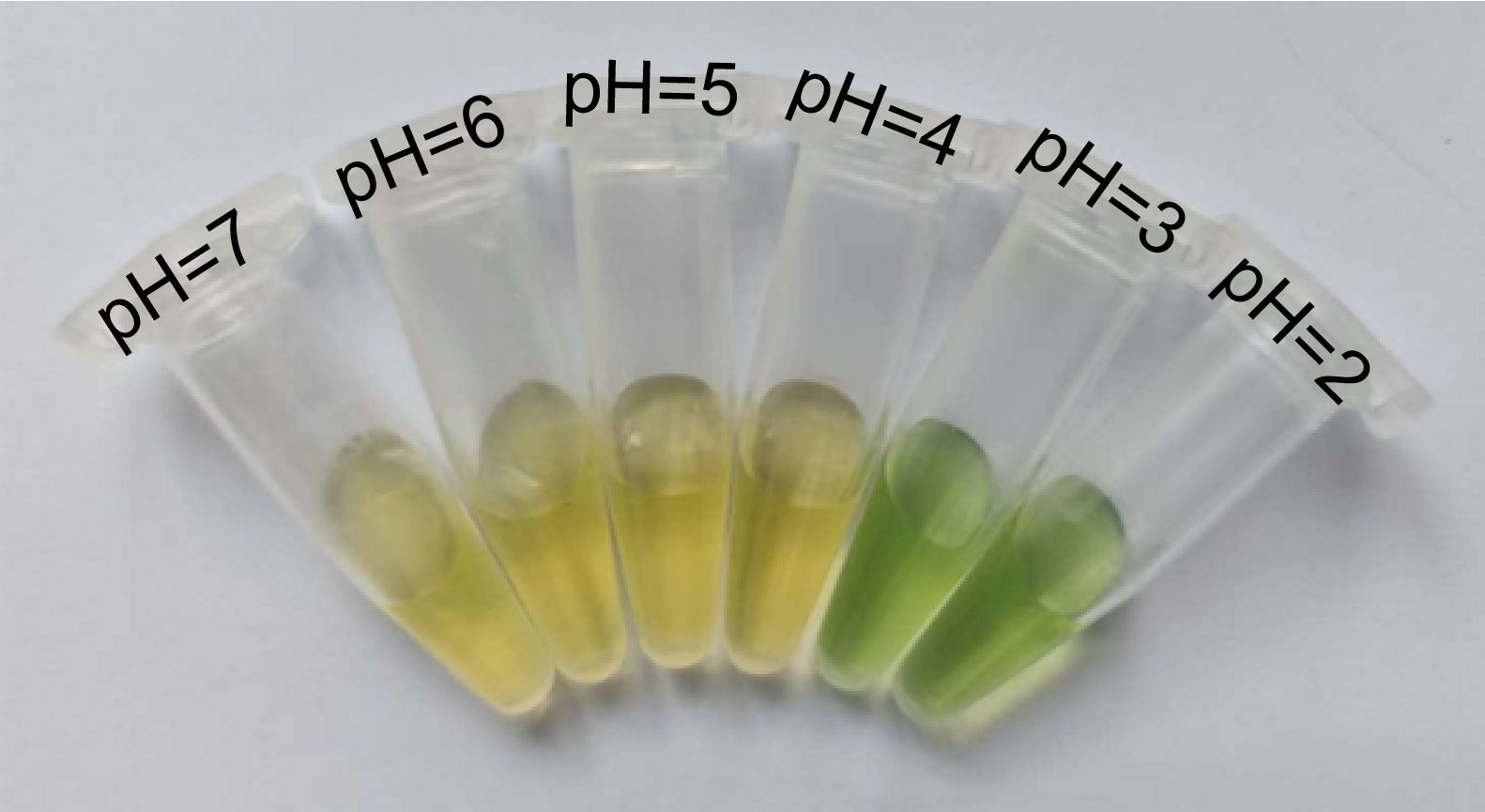


**Figure S5.** Detection of Fe²⁺ release from ENCF under different pH (7, 6, 5, 4, 3 and 2) in the presence of H_2_O_2_ (100 μM) conditions using potassium ferricyanide.


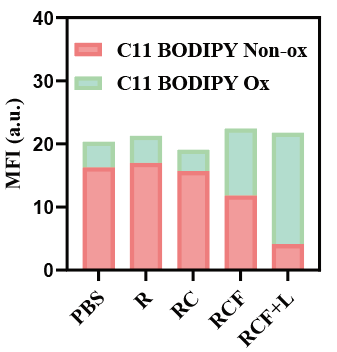


**Figure S6.** Quantitative analysis of lipid peroxidation–associated fluorescence intensity detected by C11-BODIPY^581^/^591^.


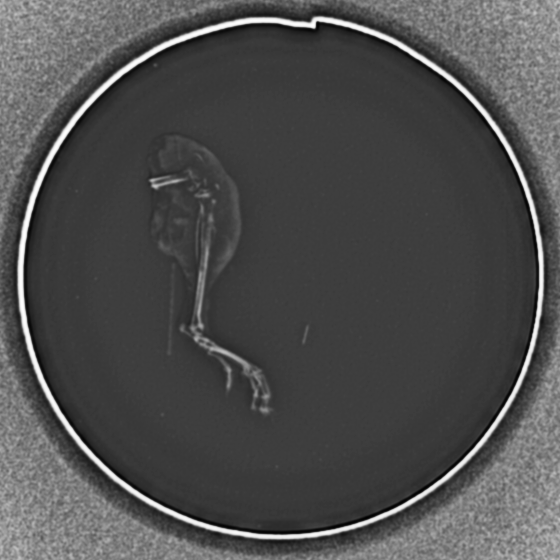


**Figure S7.** Representative X-ray images of tibial bone in PBS (untreated tumor-bearing) group.


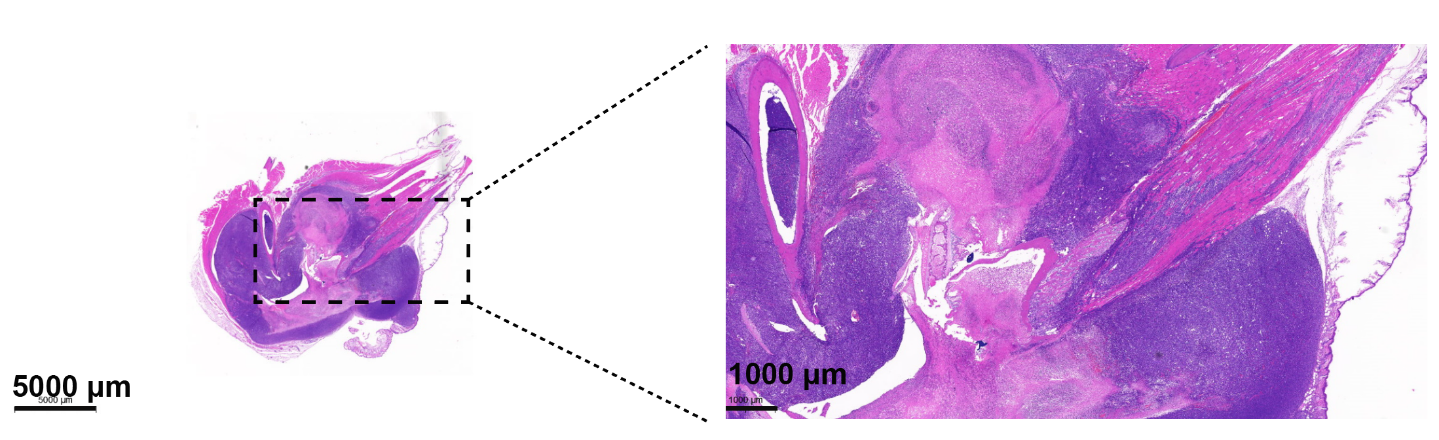


**Figure S8.** Histological H&E staining of tibial sections in the PBS group further verified tumor cell infiltration into the marrow cavity and bone destruction.


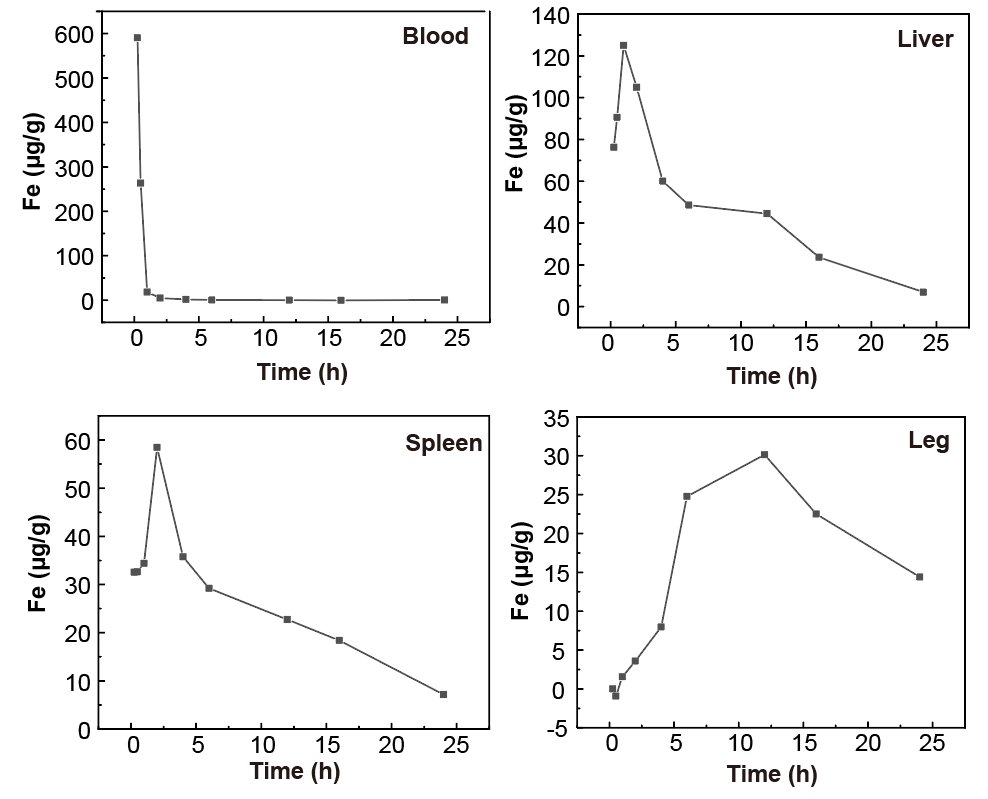


**Figure S9.** Tissue distribution of Fe after i.v. administration of the ENCF. Data were obtained by using ICP-MS to test the Fe concentration in the Blood, liver, spleen and leg.


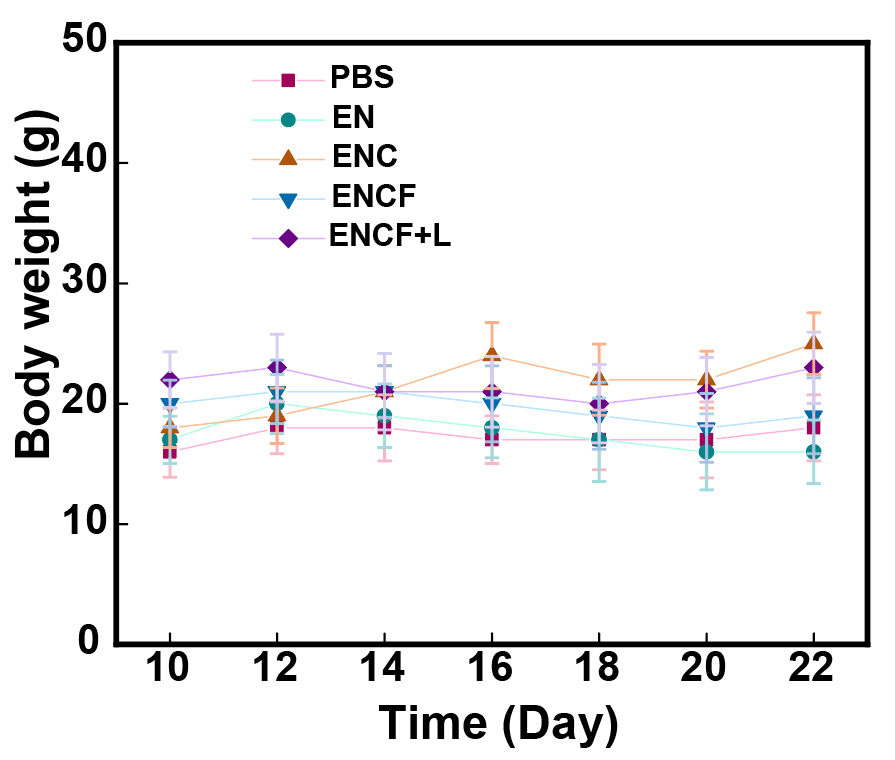


**Figure S10.** Body weight monitoring of tumor-bearing mice during treatment.


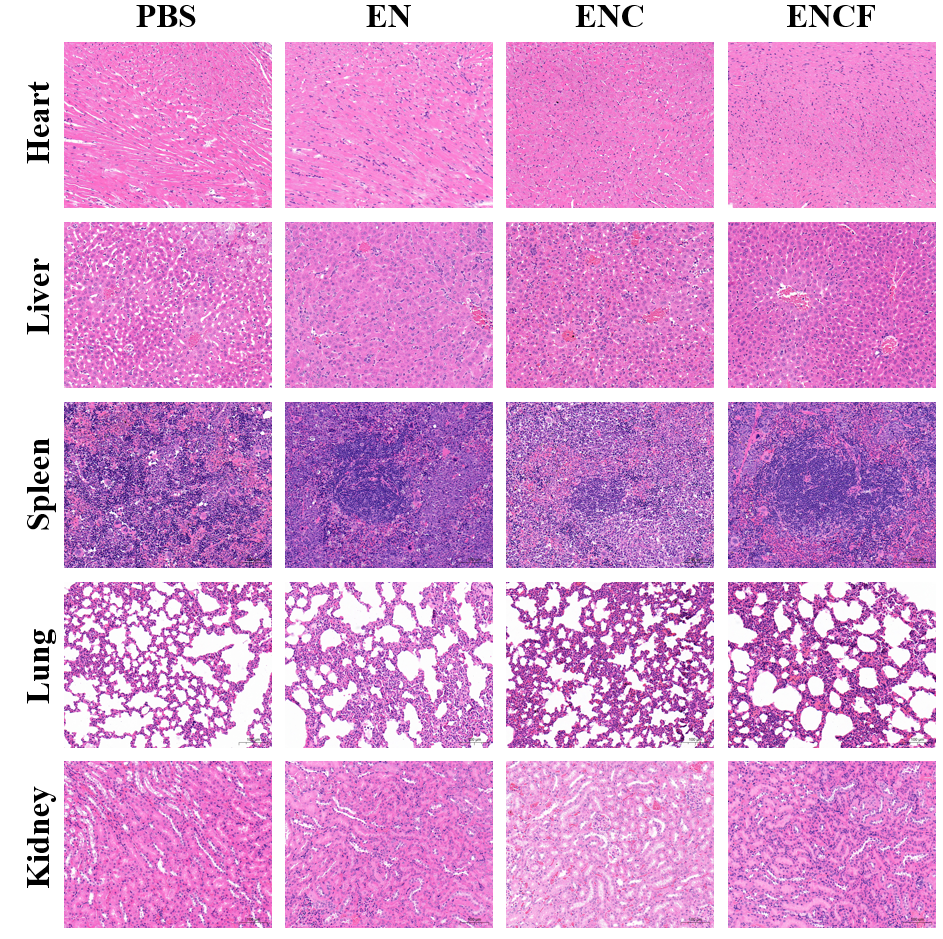


**Figure S11.** H&E staining of major organs (heart, liver, spleen, lung, kidney) from different treatment groups.


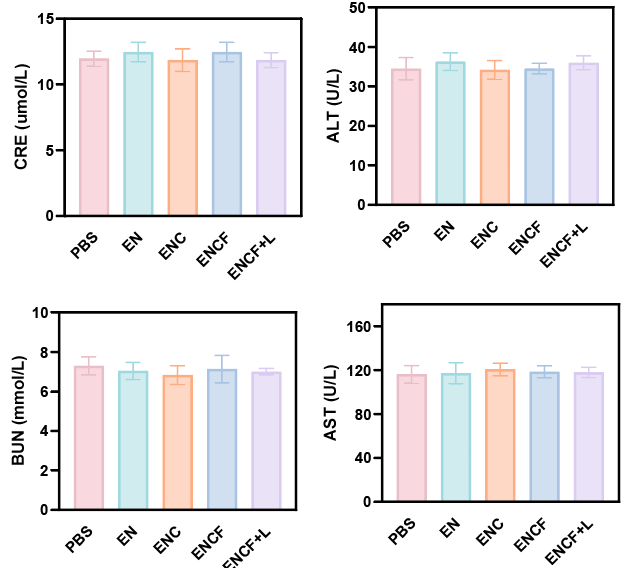


**Figure S12.** Serum levels of CRE, ALT, BUN and AST in mice treated with EN, ENC, ENCF and ENCF+L.


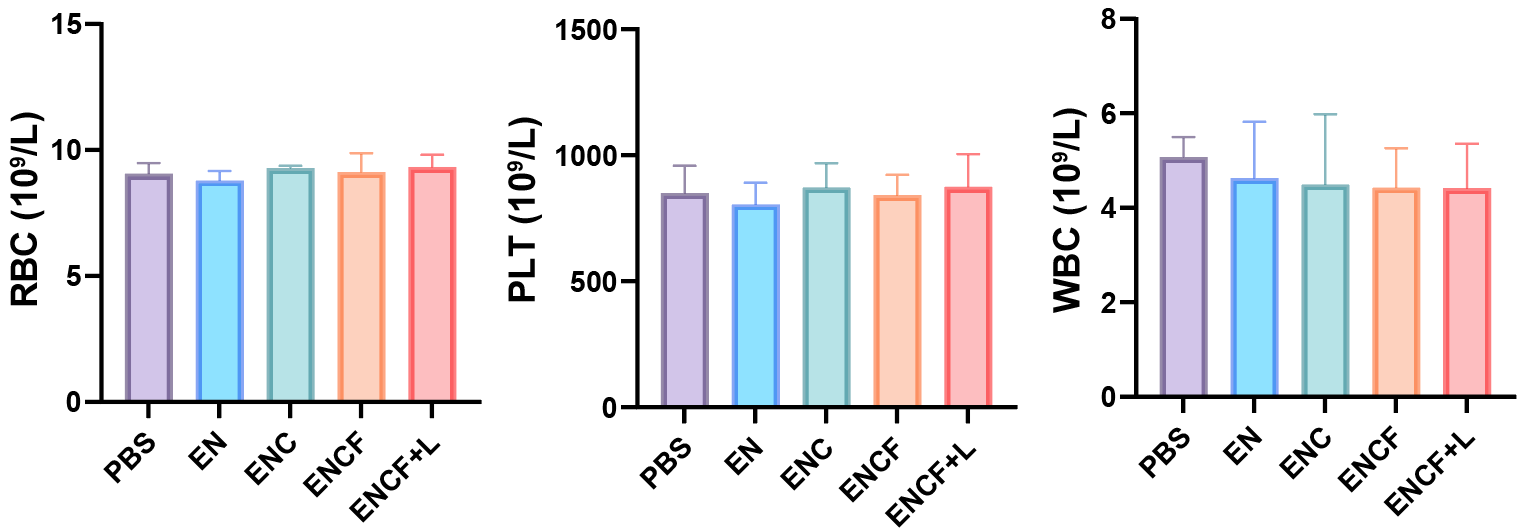


**Figure S13.** Complete blood count (CBC) analysis of ENCF-treated mice (major items: RBC, red blood cell; PLT, platelets; WBC, white blood cell)


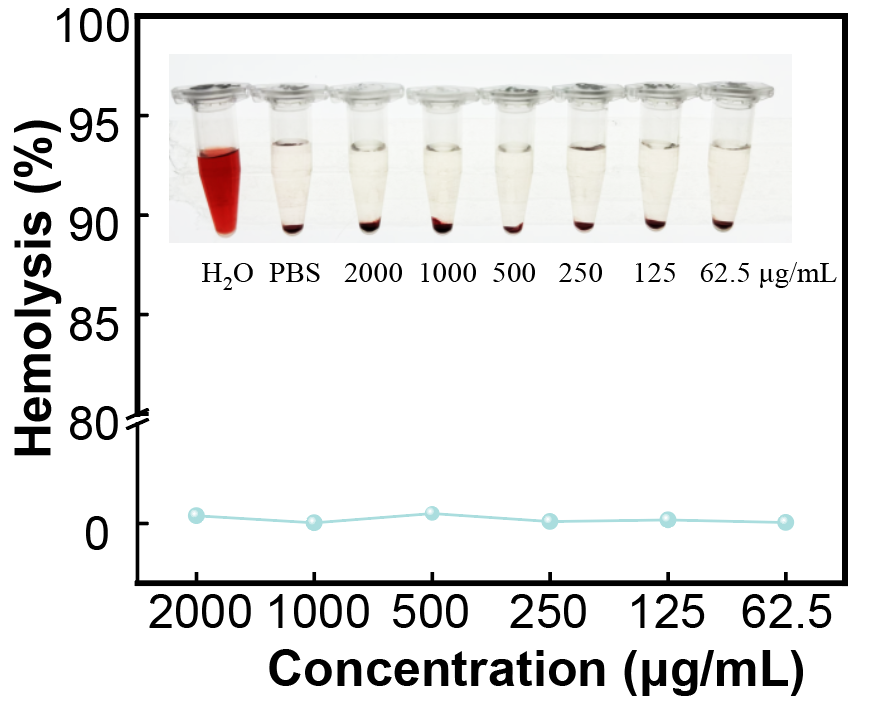


**Figure S14.** Hemolysis test of ENCF at different concentrations (62.5, 125, 250, 500, 1000 and 2000 μg/mL) in PBS.
